# Retroviral insertions contributed to the divergence of human and chimpanzee brains

**DOI:** 10.1126/sciadv.aeg5791

**Published:** 2026-10-23

**Authors:** Patricia Gerdes, Ofelia Karlsson, Raquel Garza, Diahann A. M. Atacho, PingHsun Hsieh, Bharat Prajapati, DongAhn Yoo, Chandramouli Muralidharan, Anita Adami, Carrie Davis-Hansson, Emily M. Johansson, Yogita Sharma, Laura Castilla-Vallmanya, Wiktor West, Meghna Vinod, Lucian Domitrovic, Pia A. Johansson, Jenny Johansson, Christopher H. Douse, Chandrasekhar Kanduri, Patric Jern, Evan E. Eichler, Johan Jakobsson

**Affiliations:** ^1^ Laboratory of Molecular Neurogenetics, Department of Experimental Medical Science, Wallenberg Neuroscience Center and Lund Stem Cell Center, BMC A11, Lund University, 221 84 Lund, Sweden.; ^2^ Department of Genetics, Cell Biology, and Development, Institute for Health Informatics and Bioinformatics and Computational Biology Graduate Program, University of Minnesota, Twin Cities, MN, USA.; ^3^ Department of Medical Biochemistry and Cell Biology, Institute of Biomedicine, University of Gothenburg, SE-40530 Gothenburg, Sweden.; ^4^ Department of Genome Sciences, University of Washington School of Medicine, Seattle, WA, USA.; ^5^ Laboratory of Epigenetics and Chromatin Dynamics, Department of Experimental Medical Science, Wallenberg Neuroscience Center and Lund Stem Cell Center, BMC A11, Lund University, 221 84 Lund, Sweden.; ^6^ Department of Medical Biochemistry and Microbiology, Uppsala University, Uppsala, Sweden.; ^7^ Howard Hughes Medical Institute, University of Washington, Seattle, WA, USA.

## Abstract

Over the past 5 to 7 million years, humans and chimpanzees have diverged in brain size, structure, and cognition despite high conservation of protein-coding genes. However, retroviral infections within host genomes have introduced numerous species-specific regulatory elements. Chimpanzees harbor lineage-specific PTERV1 endogenous retroviruses that are absent in humans, but their role in brain evolution has remained unclear. We performed an epigenomic analysis of PTERV1 insertions in chimpanzee neural organoids and found they are heavily DNA methylated, forming over 150 species-specific heterochromatin domains with the potential to influence gene regulation. We identified a chimpanzee-specific insertion on chromosome 19 that silences the long noncoding RNA *LINC00662* through DNA methylation spreading. CRISPR-mediated deletion of this PTERV1 restored *LINC00662* expression in chimpanzee induced pluripotent stem cells. We show that *LINC00662*, a human-specific transcript expressed during brain development, plays a role in neuronal maturation, axon outgrowth, and neural organoid development. These findings reveal a mechanism by which endogenous retroviral insertions contributed to human-chimpanzee brain divergence.

## INTRODUCTION

Among primates, the human brain stands out as the largest and most complex due in part to an increase in cortical neurons and increase in synaptic connectivity. With a size roughly three times that of our closest living evolutionary relative, the chimpanzee, it has unique cognitive capabilities (*1*). The underlying genetic divergences responsible for these differences are still poorly understood (*2*). Humans share more than 96% of protein-coding sequences with chimpanzees, making it unlikely that species-specific protein-coding variants are the sole drivers of differences in brain size and complexity (*3*). Rather, a substantial part of the genetic basis for the differences between nonhuman primate and human brains is likely to lie in the noncoding part of the genome, either as noncoding single base-pair substitutions or as structural variants (*4*–*9*). Previous studies have implicated transposable element (TE)–driven remodeling of gene regulatory networks in shaping human-specific gene expression patterns during brain development and evolution (*5*, *10*–*15*). However, the contributions of TEs to primate brain speciation remain largely unexplored.

A large proportion of primate genome sequences is made up of TEs, with one major family being endogenous retroviruses (ERVs), which have expanded throughout primate evolution. In humans and chimpanzees, at least 8% of the genome consists of ERVs, including both full-length proviruses and solo long terminal repeat (LTR) fragments (*9*, *16*, *17*). Most primate ERVs are believed to have originated from exogenous retroviral integration events in the germ line. These ERVs then expanded through reinfection or intercellular retrotransposition and were subsequently fixed in the genome, probably through population bottlenecks (*18*). Comparative analyses of closely related primate genomes have revealed substantial differences in ERV composition between species, which provides a genomic fossil of ancient retroviral pandemics. These differences demonstrate that ERVs represent a rich source of species-specific structural variants (*9*, *19*, *20*). Furthermore, ERVs have the potential to affect genomes by altering gene regulatory networks. For example, ERVs harbor promoter and enhancer sequences that can affect the expression patterns of nearby genes in cis (*21*, *22*). In addition, ERVs are preferential sites for DNA methylation, which may influence the epigenetic status of their integration sites and silence genes in the vicinity (*23*–*26*). This capacity of ERVs to influence host gene expression has led to the idea that ERVs may be actively involved in speciation by contributing to species-specific gene regulatory networks (*27*–*30*).

The rate of ERV insertions into the human genome has decreased during the most recent phase of human evolution. After the chimpanzee-hominoid split about 5 to 7 million years ago, only a small number of HERV-K elements integrated and became fixed in the human genome (*31*–*33*). However, this lack of a major burst of ERV integrations into the genome is unique for humans among African great apes (*34*). The chimpanzee genome was recently invaded by a retrovirus family, *Pan troglodytes* endogenous retrovirus 1 (PTERV1), which is completely absent from the human genome. PTERV1 generated at least 150 fixed insertions, making it one of the most abundant ERV families in the chimpanzee genome (*9*, *35*, *36*). These PTERV1 insertions are thought to have originated from infections that occurred in the ancestors of African apes about 5 to 7 million years ago (*36*). PTERV1 elements are found in chimpanzees, bonobos, and gorillas; however, only a single orthologous insertion has been identified between chimpanzee and gorilla lineages, suggesting independent expansion in these species (*9*) (Fig. 1A). Notably, the absence of PTERV1 insertions from the human genome suggests that either the human and chimpanzee lineages did not occupy the same habitat at the time or that germline-competent cells of the human ancestor were resistant to this infection. In line with this latter idea, each primate species encodes a TRIM5α protein with different antiviral specificity and it has been shown that the human version of TRIM5α prevents infection of human cells with PTERV1 (*37*). The endoretroviral pandemic underlying the integration of PTERV1 sequences into the ancestral chimpanzee genome is likely to have had a major negative impact on the fitness and size of the population at that time and could have contributed to the speciation between the human and chimpanzee lineages by changing population structure and habitat. In addition, a more indirect but long-lasting effect of this retroviral pandemic could be that fixed PTERV1 insertions affected gene regulatory networks, thereby acting as a source of genetic divergence that could contribute to the speciation between human and chimpanzee ancestors. However, experimental evidence for this hypothesis is thus far lacking.

**Fig. 1.**
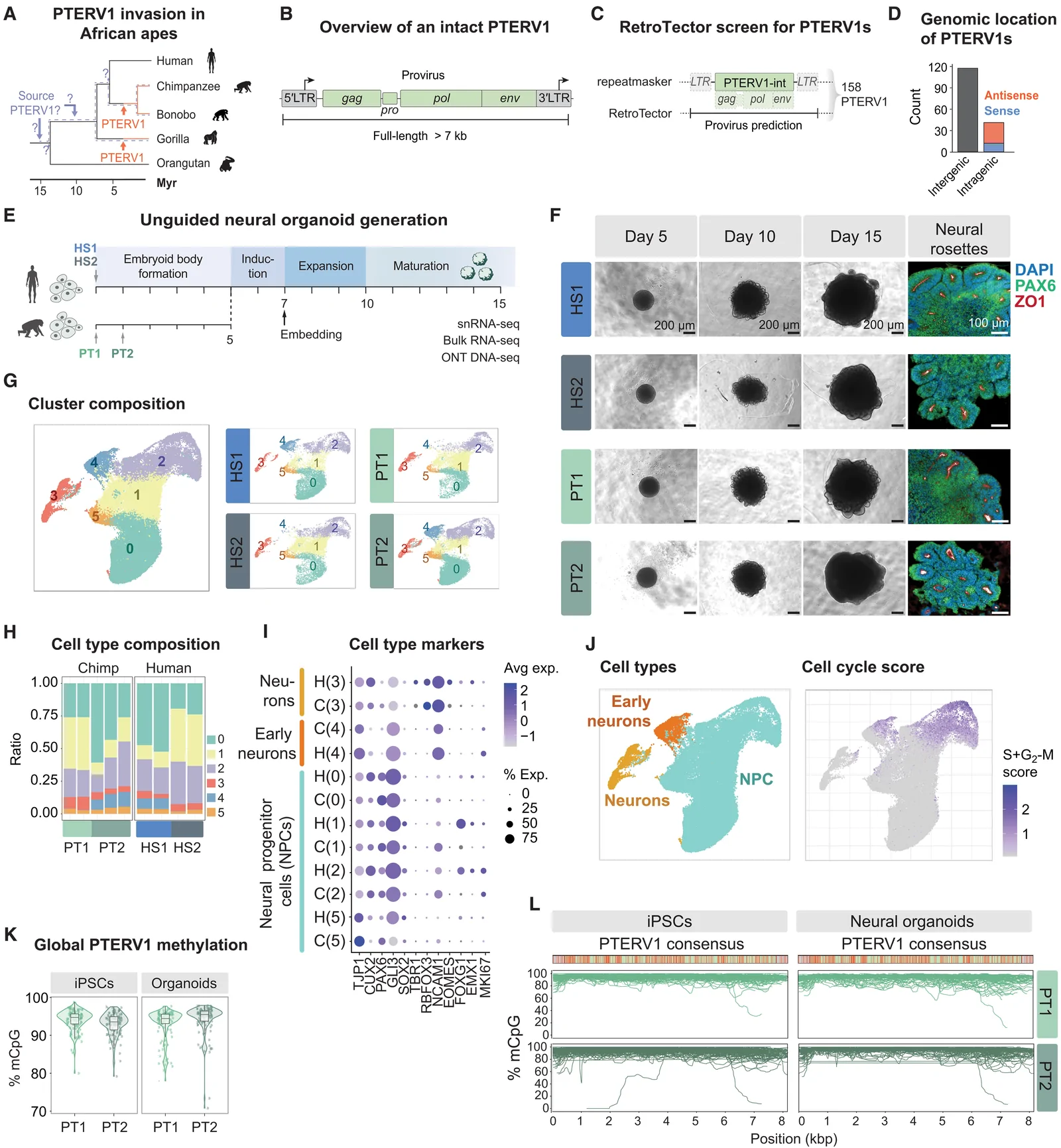
PTERV1 insertions form chimpanzee-specific methylated heterochromatin domains in neural organoids. (**A**) Phylogenetic tree showing the estimated PTERV1 infection and colonization of Great Ape genomes. Myr, million years. (**B**) Structure of a PTERV1 element, with two 400- to 600-bp LTRs (gray boxes) flanking retroviral genes (*gag*, *pro*, *pol*, and *env*; green boxes); black arrows indicate the promoter function. (**C**) Schematic of the RetroTector screen and analysis of PTERV1 proviruses in the chimpanzee genome (CLINT_PTRv2/panTro6). (**D**) PTERV1 integration sites in the chimpanzee genome, classified as inter- or intragenic using the panTro6 RefSeq transcript annotation; orientation was defined by 5′ to 3′ synthesis and classified relative to transcript orientation. (**E**) Timeline for unguided neural organoid generation from human and chimpanzee iPSCs; colors indicate media and stages. PT2 organoids have a 1-day shorter EB stage. DNA-seq, DNA sequencing. (**F**) Bright-field images of human (HS1 and HS2) and chimpanzee (PT1 and PT2) organoids at days 5, 10, and 15. Scale bars, 200 μm. Right: Immunofluorescence images of day 15 neural rosettes stained for PAX6 (green), ZO1 (red), and DAPI (blue). Scale bars, 100 μm. (**G**) UMAPs colored by nuclei clusters found in day 15 neural organoids with all nuclei (left) and split per cell line (right). (**H**) Bar plot showing the cluster composition per sample. (**I**) Expression of markers across species. Color showing the average expression in cluster, and dot size showing the percentage of nuclei in cluster where the gene was detected. (**J**) Left: UMAP colored by cell types. Right: UMAP colored by cell cycle score (sum of module scores of genes associated to the S phase and G_2_-M phase. Seurat::CellCycleScoring). (**K**) PTERV1 CpG methylation ascertained by ONT sequencing of chimpanzee iPSCs and day 15 neural organoids. Dots represent PTERV1 loci. (**L**) Composite FL-PTERV1 methylation profiles. Schematic of the FL-PTERV1 consensus is shown at the top with CpG dinucleotides indicated in orange. kbp, kilo–base pairs. Created in BioRender. Gerdes, P. (2026) https://BioRender.com/dp52mbp.

In this study, we conducted a global epigenomic analysis of PTERV1 insertions in chimpanzee neural organoids. We found that these elements are heavily covered by DNA methylation, representing species-specific heterochromatin domains. We identified a PTERV1 insertion on chimpanzee chromosome 19 that affects gene regulatory networks in brain development through the transcriptional repression of the long noncoding RNA (lncRNA) *LINC00662*, via accumulation of DNA methylation. We were able to restore *LINC00662* expression in chimpanzee cells using CRISPR-based deletion of this PTERV1 in chimpanzee induced pluripotent stem cells (iPSCs). We found that *LINC00662* is a human-specific transcript expressed in the developing brain. It is localized in the cytoplasm, where it plays a role in the maturation of neurons, thereby influencing the development of neural organoids. In summary, this study describes how retroviral insertions contributed to the functional divergence of the human and chimpanzee brain, providing a mechanism by which retroviral infections influenced primate brain speciation.

## RESULTS

### PTERV1s are covered by DNA methylation in chimpanzee iPSCs and neural organoids

A full-length PTERV1 is typically 7 to 10 kb in length and encodes the retroviral genes *gag*, *pro*, *pol*, and *env*, which are flanked by two LTRs that are identical at the time of integration and usually 400 to 600 base pairs (bp) long (*35*, *36*) (Fig. 1B). Because of the fragmented RepeatMasker annotation of ERVs in reference genomes, which splits ERVs into LTRs and internal regions, we used the RetroTector software (*38*) to screen the chimpanzee reference genome (CLINT_PTRv2/panTro6) for PTERV1 proviruses. We found 158 PTERV1 elements which ranged in size from 2 to 14 kb with an average length of 7.5 kb (Fig. 1C and data S1). In agreement with previous analysis (*9*), we found that most of these PTERVs were intergenic and integrated preferentially in antisense direction when located within genes, indicating purifying selection (Fig. 1D).

Comparative evolutionary analyses of hominoid brain development have long been hampered by the lack of availability of chimpanzee tissue from early developmental periods. However, with the generation of iPSCs of chimpanzee origin, it has now become possible to develop in vitro cell culture models to study hominoid brain development (*39*–*43*). To investigate the epigenetic status of individual PTERV1 elements and their potential effect on gene regulatory networks in brain development, we optimized a protocol to generate chimpanzee and human unguided neural organoids under near-identical conditions to obtain highly comparable organoids for the two species (Fig. 1E). We observed similar size and morphology between chimpanzee and human organoids up to 15 days of differentiation using iPSCs from two individuals of each species (fig. S1A). At this developmental stage, organoids from both species showed similar neural rosette formation typical for unguided neural organoids, as monitored with immunohistochemical staining for PAX6, a neural identity marker, ZO1, a tight junction protein (*44*, *45*) (Fig. 1F), SOX2, a neural progenitor cell (NPC) marker, and phospho-Histone H3 (pH3), a marker of mitotic cells (fig. S1B). Single-nucleus RNA sequencing (snRNA-seq) analysis confirmed a similar cell type composition of the chimpanzee and human organoids, with most cells being NPCs or early-born neurons at day 15 of neural organoid development (Fig. 1, G to J).

To investigate the epigenetic status of PTERV1s in chimpanzee iPSCs and unguided neural organoids, we performed genome-wide CpG DNA methylation profiling using long-read DNA sequencing from Oxford Nanopore Technologies (ONT) as this approach allows the analysis of DNA methylation patterns of individual PTERV1 loci at single base-pair resolution. This analysis revealed that almost all PTERV1 loci in iPSCs and day 15 unguided neural organoids are completely methylated (Fig. 1, K and L). Because PTERV1 integrations are absent from the human genome, this result demonstrates that the PTERV1 insertions represent 158 chimpanzee-specific local heterochromatin domains.

### The expression of *LINC00662* in chimpanzee is silenced by a PTERV1 insertion

We hypothesized that methylated PTERV1 insertions might affect gene expression in cis through the spread of local heterochromatin. Therefore, we intersected PTERV1 insertions with genes that have a homolog in both species (Fig. 2A), considering only candidates fully contained within gene bodies. Among the 45 genes that intersected with a PTERV1 insertion, we found 10 genes, including both protein-coding and noncoding transcripts, showed differential expression when comparing bulk RNA sequencing (RNA-seq) data from human and chimpanzee unguided neural organoids (|log_2_FoldChange| > 1; *P*_adj_ < 0.05, Wald test, DESeq2) (Fig. 2B); most were intronic (table S1). The top candidate of these genes was *LINC00662*, an lncRNA that is highly expressed in human unguided neural organoids but completely silenced in chimpanzee organoids (Fig. 2B).

**Fig. 2.**
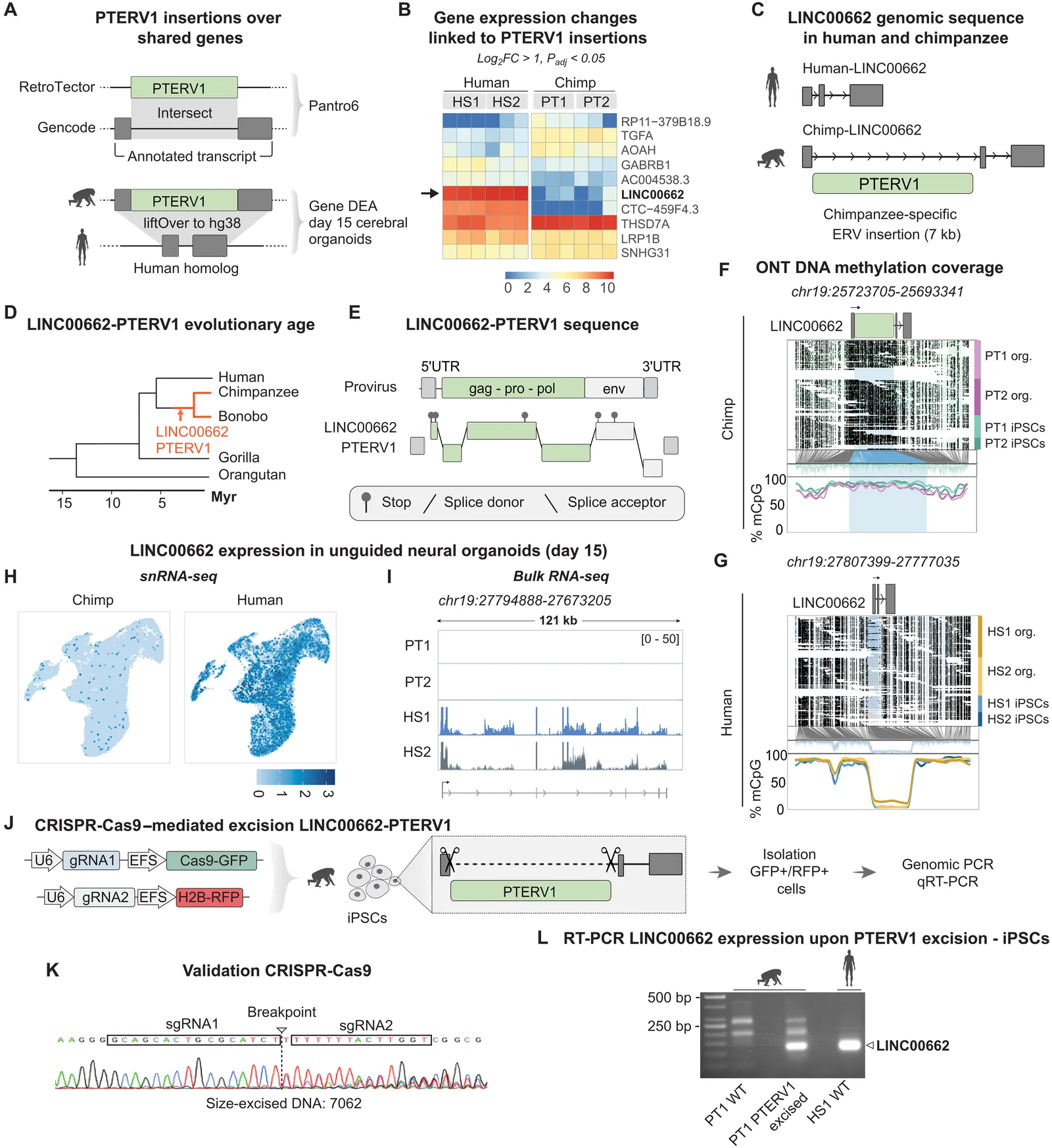
A chimpanzee-specific PTERV1 insertion silences the lncRNA *LINC00662* through DNA methylation spreading. (**A**) Workflow for comparing PTERV1-containing transcripts and human homologs in day 15 unguided neural organoids. DEA, differential expression analysis. (**B**) Expression of transcripts with chimpanzee PTERV1 insertions in day 15 human HS1 (*n* = 3) and HS2 (*n* = 3) and chimpanzee PT1 (*n* = 3) and PT2 (*n* = 3) organoids by bulk RNA-seq. Results are shown as log_2_FoldChange (Log_2_FC) > 1 and *P*_adj_ < 0.05. (**C**) Schematic of the human and chimpanzee *LINC00662* locus indicating the chimpanzee PTERV1 insertion. (**D**) Phylogenetic tree indicating the estimated evolutionary age of *LINC00662*-PTERV1. (**E**) PTERV1 schematic and three-frame chain view of *LINC00662*-PTERV1 from RetroTector, showing stop codons and frameshifts. 5′UTR, 5′ untranslated region; 3′UTR, 3′ untranslated region. (**F**) ONT DNA methylation profile of the *LINC00662*-PTERV1 locus. The top panel shows the PTERV1 insertion in sense orientation in intron 1 of chimpanzee *LINC00662*. The second panel shows ONT read alignments from PT1 and PT2 iPSCs and iPSC-derived neural organoids. Unmethylated CpGs are colored by sample; methylated CpGs are black. The third panel indicates CpG positions, including PTERV1 CpGs (blue shading). The fourth panel shows the methylated CpG fraction. (**G**) As in (F), but for the human *LINC00662* locus without PTERV1. Samples are human iPSC lines HS1 and HS2 and iPSC-derived unguided neural organoids (*n* = 2). (**H**) *LINC00662* expression in human and chimpanzee iPSC-derived unguided neural organoids at day 15 by snRNA-seq. (**I**) Genome browser tracks showing the *LINC00662* expression in human and chimpanzee day 15 iPSC-derived unguided neural organoids by bulk RNA-seq. (**J**) Workflow for CRISPR-Cas9–mediated excision of PTERV1 in chimpanzee PT1 iPSCs. (**K**) Chromatogram showing sequencing results after the CRISPR-Cas9–mediated excision of *LINC00662*-PTERV1 in PT1. (**L**) RT-PCR products from chimpanzee iPSCs, chimpanzee PTERV1-excised iPSCs, and human iPSCs separated on an agarose gel. Expected human *LINC00662* and chimpanzee PTERV1-excised *LINC00662* amplicons are indicated by a black arrow. Created in BioRender. Gerdes, P. (2026) https://BioRender.com/vnqntmm.

Pairwise sequence alignment of the human and chimpanzee *LINC00662* genome sequences revealed that the chimpanzee locus contains a 6915–nucleotide (nt) PTERV1 insertion integrated into the negative strand in sense within the first intron of *LINC00662* (Fig. 2C). We found no other major differences between the human and chimpanzee genome at this locus (fig. S2A), indicating that the PTERV1 insertion is likely to be the underlying cause for the transcriptional differences. When examining telomere-to-telomere (T2T) great ape and macaque assemblies (*3*, *46*), we found that the *LINC00662*-PTERV1 insertion is only present in the chimpanzee and bonobo genomes. This suggests that the PTERV1 insertion occurred between the splits of the human and chimpanzee lineages and the chimpanzee and bonobo lineages, which occurred between 1.5 and 5 million years ago (Fig. 2D). In gorilla and macaque, the ∼300-kb segment containing *LINC00662* is entirely absent. Orangutans do not have the PTERV1 insertion but instead have an 8.8-kb LINE-1 retrotransposon insertion upstream of *LINC00662* (fig. S2, A to C, and table S2). Analysis of the 5′ and 3′ LTR divergence of the flanking LTR elements of the *LINC00662*-PTERV1 showed a low divergence (1.2% mutation rate), confirming the young evolutionary age of this retroviral insertion (Fig. 2D). Structural analysis of the *LINC00662*-PTERV1 provirus sequence, using the RetroTector software, confirmed that it is of gamma-retrovirus origin and contains the retroviral genes *gag*, *pro*, *pol*, and *env* (Fig. 2E). The gamma-retrovirus gag-pro-pol polyprotein is normally expressed in a continuous reading frame with a read-through stop after *gag*. The *LINC00662*-PTERV1 provirus *gag-pro-pol* contains multiple inactivating frameshifts and stop codons. In addition, the *LINC00662*-PTERV1 *env* displays frameshifts and stop codons (Fig. 2E). In summary, the *LINC00662*-PTERV1 sequence diverges only slightly from the PTERV1 consensus sequence, but it is likely replication deficient due to frameshifts and premature stop codons.

As PTERV1 insertions are highly methylated (Fig. 1, K and L), we hypothesized that the *LINC00662*-PTERV1 silences the *LINC00662* promoter by introducing a local heterochromatin domain. To investigate the epigenetic status of the *LINC00662* locus in humans and chimpanzees, we used the ONT DNA sequencing data from iPSCs and neural organoids. In chimpanzee iPSCs and neural organoids, the *LINC00662*-PTERV1 insertion was highly methylated, as were the flanking genomic regions (Fig. 2F). On the contrary, we found low methylation coverage across the *LINC00662* locus in human iPSCs and neural organoids (Fig. 2G). These results suggest that the PTERV1 insertion in the chimpanzee *LINC00662* locus attracts DNA methylation that spreads into the flanking genome, contributing to the differences in DNA methylation patterns at this locus between humans and chimpanzees.

To further dissect the transcriptional divergence of *LINC00662*, we used our neural organoid bulk RNA-seq and snRNA-seq data, which confirmed that *LINC00662* was exclusively expressed in human samples (Fig. 2, H and I). *LINC00662* was expressed throughout the different cell populations in unguided neural organoids, ruling out the possibility that the human-specific expression was due to a specific subpopulation in the human samples. We used our previously published CUT&RUN epigenomic profiling data of human and chimpanzee forebrain NPCs (fbNPCs) grown in 2D (*6*), which showed a notable human-specific enrichment of the transcription-related epigenetic mark H3K4me3 over the promoter of *LINC00662*, confirming that the observed differences in RNA levels were due to differences in transcriptional activity (fig. S2D). To further verify the human-specific expression of *LINC00662*, we analyzed publicly available RNA-seq data of human, chimpanzee, orangutan, and rhesus macaque cortical organoids as well as of human, chimpanzee, and bonobo NPCs (*47*, *48*). These datasets revealed expression of *LINC00662* in human but not chimpanzee, orangutan, rhesus macaque, or bonobo neural cell models (fig. S2, E and F). To further verify that our results were not limited to the two chimpanzee individuals used to derive the iPSCs in this study, a dataset of iPSCs of eight additional individuals confirmed human-specific expression of *LINC00662* (*49*) (fig. S2G). Furthermore, GTEx data (*50*) showed that *LINC00662* is broadly expressed across human tissues, with relatively higher expression in brain and testis, indicating a potential functional role in the human brain (fig. S2H).

To investigate mechanistically whether the PTERV1 insertion is responsible for the transcriptional silencing of *LINC00662*, we used CRISPR-Cas9 gene editing to excise it in chimpanzee iPSCs. Two guide RNAs (gRNAs) were designed to target the flanking regions of the *LINC00662*-PTERV1 and cloned into lentiviral vectors that also express Cas9 (Fig. 2J). Amplicon sequencing of chimpanzee iPSCs transduced with the CRISPR-Cas9 vector confirmed the excision of the ∼7-kb PTERV1 (Fig. 2K). Reverse transcription polymerase chain reaction (RT-PCR) analysis showed that, upon excision of PTERV1, *LINC00662* was expressed in chimpanzee iPSCs (Fig. 2L). Thus, this experiment demonstrates that the *LINC00662*-PTERV1 insertion mediates transcriptional silencing of *LINC00662* in chimpanzees as PTERV1 excision restores *LINC00662* expression in chimpanzee cells.

### *LINC00662* is a trans-acting lncRNA expressed during human brain development

To investigate *LINC00662* expression during human brain development in vivo, we analyzed publicly available bulk RNA-seq data from different stages of the developing and adult human brain (*51*, *52*). We found that *LINC00662* expression peaks in early brain development at around 10 to 12 weeks postconception (PC), followed by gradual down-regulation in adulthood (Fig. 3A). snRNA-seq analysis of human fetal forebrain development using our previously published dataset (*5*) showed that *LINC00662* is expressed throughout the developing human forebrain at weeks 7 and 11 weeks PC (Fig. 3B). We further confirmed that *LINC00662* is expressed in human fetal forebrain development at 7 and 11 weeks PC using our previously published bulk RNA-seq and H3K4me3 CUT&RUN dataset (*5*). In addition, ONT direct long-read RNA-seq showed that *LINC00662* expression in the fetal human brain at 9 and 10 weeks PC starts at the transcription start site (TSS), which overlaps with the H3K4me3 peak, and that it is a spliced lncRNA that exists in several different isoforms due to alternative transcription stop sites (Fig. 3C). ONT long-read DNA sequencing revealed low DNA methylation coverage across the *LINC00662* locus in human fetal brain tissue at weeks 7 and 11 PC (Fig. 3D). Together, these results demonstrate that *LINC00662* is a noncoding transcript expressed during early human brain development.

**Fig. 3.**
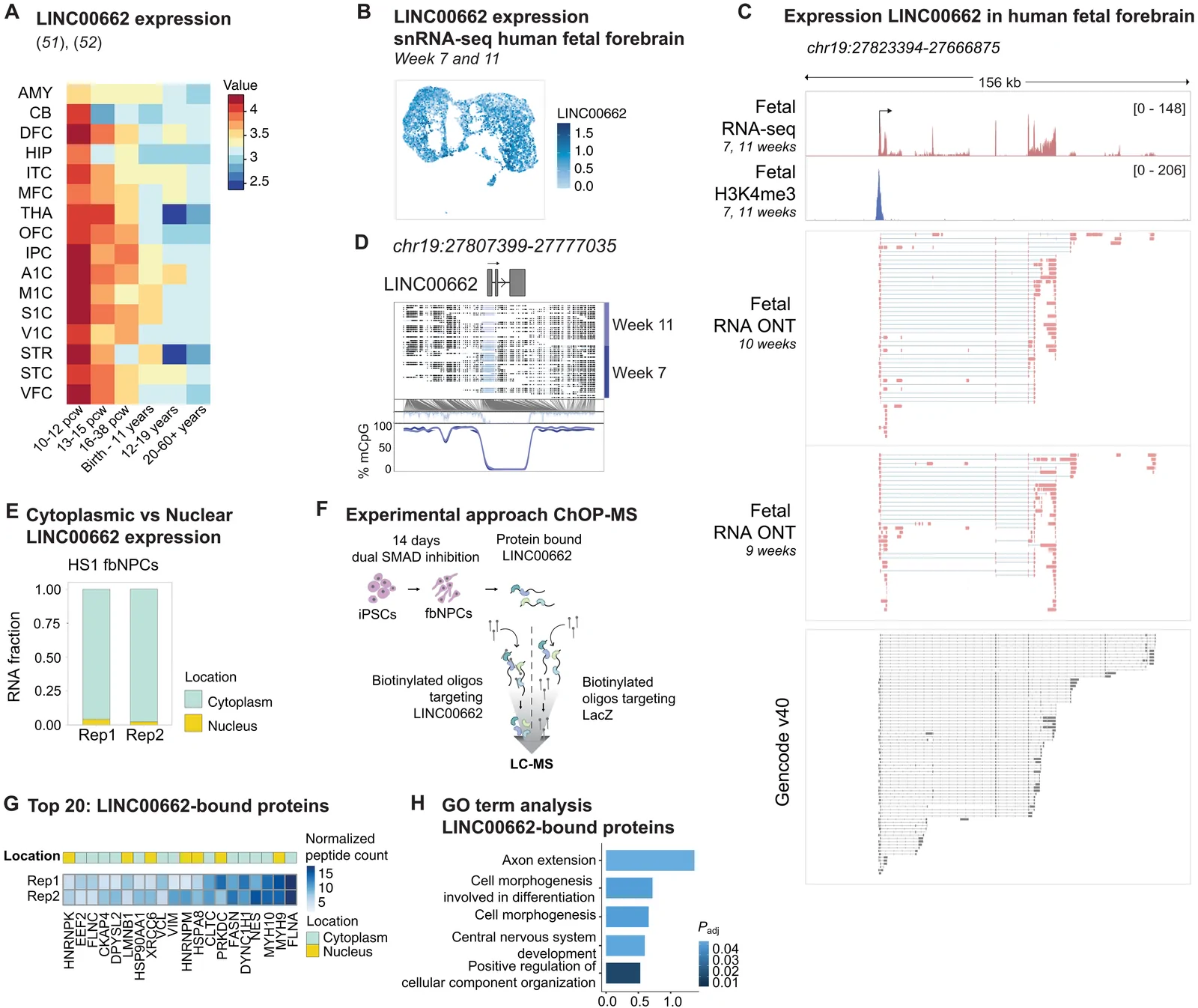
*LINC00662* is a cytoplasmic human lncRNA expressed during brain development and associated with neurite-related proteins. (**A**) Bulk RNA-seq showing the *LINC00662* expression in human fetal and adult brain samples presented as Log_2_ CPM. Data from Miller *et al.* (*52*) and Cardoso-Moreira *et al.* (*51*). AMY, amygdala; CB, cerebellar cortex; DFC, dorsolateral prefrontal cortex; HIP, hippocampus; ITC, inferior temporal cortex; MFC, medial prefrontal cortex; THA, mediodorsal thalamus; OFC, orbital prefrontal cortex; IPC, posterior inferior parietal cortex; A1C, primary auditory cortex; M1C, primary motor cortex; S1C, primary somatosensory cortex; V1C, primary visual cortex; STR, striatum; STC, superior temporal cortex; VFC, ventrolateral prefrontal cortex. (**B**) *LINC00662* expression in human fetal forebrain samples at different gestational ages. (**C**) Genome browser tracks showing the *LINC00662* expression in human fetal forebrain by bulk RNA-seq (top), H3K4me3 CUT&RUN (middle), and direct RNA ONT reads (bottom). (**D**) ONT DNA methylation profile of the *LINC00662* locus in two fetal forebrain samples. The top panel shows *LINC00662* in the human genome. The second panel shows ONT read alignments. Unmethylated CpGs are colored by sample; methylated CpGs are black. The third panel indicates CpG positions. The fourth panel shows the methylated CpG fraction. (**E**) Subcellular localization of *LINC00662* in human HS1 fbNPCs by qRT-PCR of cytosolic and nuclear RNA fractions (*n* = 2, Rep1 and Rep2). (**F**) ChOP-MS workflow for identifying *LINC00662*-bound proteins in human fbNPCs by LC-MS. (**G**) Heatmap showing normalized values of the top 20 *LINC00662*-bound proteins and their cellular localization in human fbNPCs (*n* = 2, Rep1 and Rep2). (**H**) GO analysis of *LINC00662*-bound proteins using StringDb v11.5. Created in BioRender. Gerdes, P. (2026) https://BioRender.com/q5reo0b.

LncRNAs act in cis by influencing the expression or chromatin state of nearby genes or in trans by executing functions throughout the cell, for example in the cytoplasm (*53*). To investigate the cellular localization of *LINC00662*, we performed a cellular fractionation assay in iPSC-derived human fbNPCs, which revealed an almost exclusive presence of *LINC00662* transcripts in the cytoplasmic fraction (Fig. 3E), indicating that *LINC00662* acts in trans. Because many lncRNAs act as protein-binding RNAs, we determined the protein interactome of *LINC00662* using chromatin oligo affinity purification followed by shotgun proteomics (ChOP-MS). Using targeted biotinylated oligonucleotides, we pulled down proteins bound by LINC00662, which we characterized using liquid chromatography–mass spectrometry (LC-MS) (Fig. 3F). As a negative control we used oligonucleotides targeting *lacZ*, representing a sequence not found in the human genome. The ChOP-MS results confirmed that *LINC00662* mainly interacts with proteins found in the cytoplasm, further indicating that this lncRNA acts in trans (Fig. 3G). Gene ontology (GO) term analysis of the *LINC00662*-bound proteins indicated an enrichment of terms related to axon extension and neural development (Fig. 3, G and H). For example, proteins that are in complex with *LINC00662* include FLNA, MYH10, NES, VIM, and DPYSL2. These proteins are all related to the cytoskeleton and are well-documented examples of proteins that are linked to neurite extension and brain development (*54*–*61*) (Fig. 3G).

Together, these results suggest that *LINC00662* may play an important role in the cytoplasm in neural progenitors by interacting with proteins associated with the cytoskeleton and neurite extension.

### *LINC00662* plays a role in neurite extension in neural organoids

The *LINC00662*-protein interactome analysis indicated a role in neurite extension during brain development. To investigate the functional relevance of *LINC00662*, we developed a CRISPRi strategy to silence its expression in neural organoids. We designed two gRNAs to target unique genomic locations in the vicinity of the TSS and coexpressed these with a Krüppel-associated box (KRAB) transcriptional repressor domain fused to catalytically dead Cas9 (KRAB-dCas9) (Fig. 4A). Lentiviral transduction of human iPSCs resulted in efficient, almost complete silencing of *LINC00662* in iPSCs using both gRNAs (Fig. 4B and fig. S3A).

**Fig. 4.**
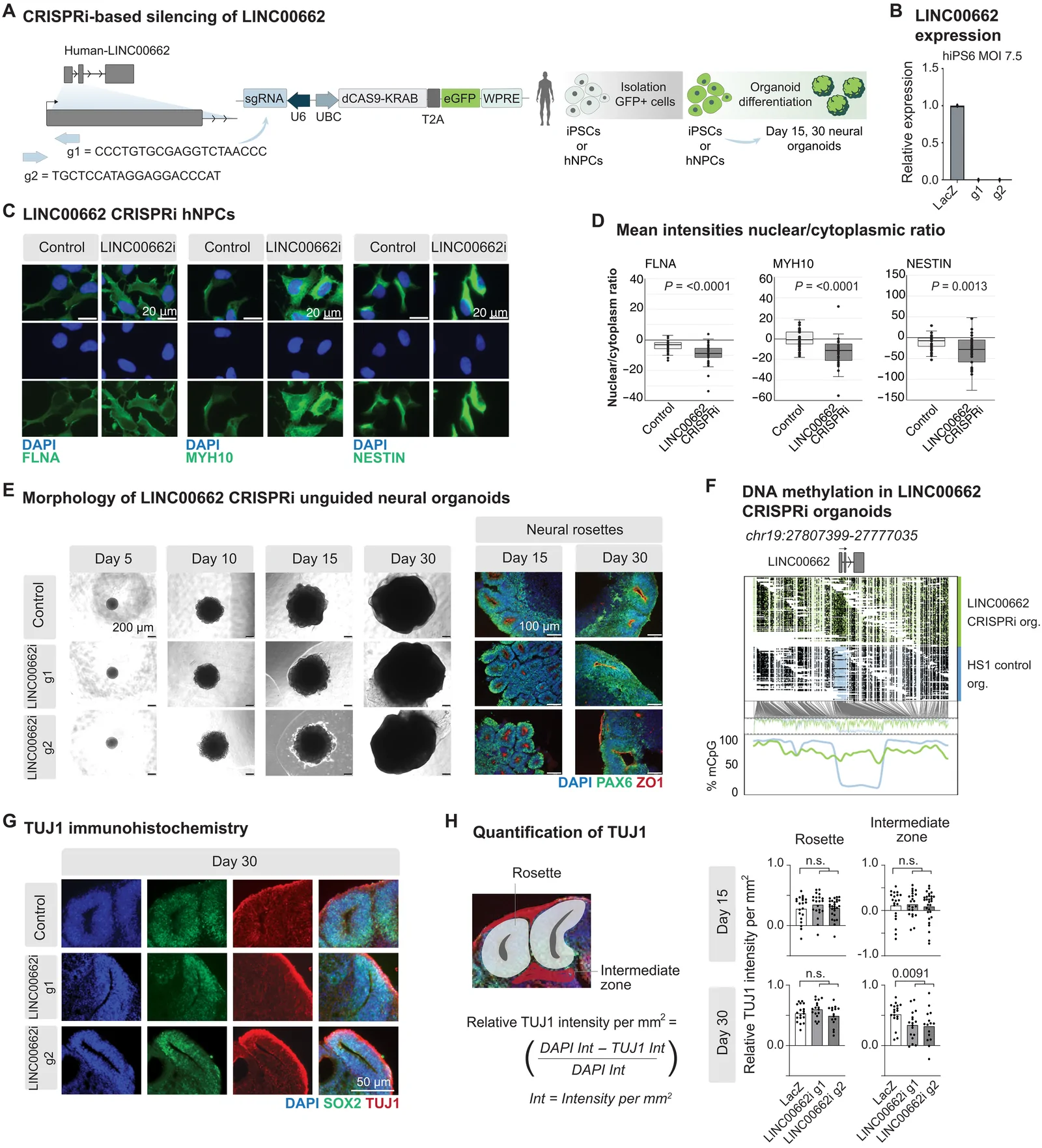
Loss of *LINC00662* impairs neurite-associated maturation in human neural organoids. (**A**) Schematics of *LINC00662*-CRISPRi design in human iPSC-derived unguided neural organoids. (**B**) qRT-PCR analysis of the *LINC00662* expression in iPSC: control (*lacZ*) and *LINC00662*-CRISPRi (gRNA1 and gRNA2). (**C**) Immunocytochemistry showing DAPI (blue) and FLNA, MYH10, or NESTIN (green) in control and *LINC00662*-CRISPRi hNPCs. Scale bars, 20 μm. (**D**) Nuclear-to-cytoplasmic ratios of FLNA, MYH10, and NESTIN in control and *LINC00662*-CRISPRi hNPCs. Ratios were calculated per cell using mean fluorescence intensities in the nucleus and cytoplasm. Box-and-whisker plots show the median and range. Unpaired *t* test with Welch’s correction. (**E**) Left: Bright-field images of iPSC-derived *LINC00662*-CRISPRi neural organoids. Scale bars, 200 μm. Right: Immunohistochemistry of day 15 and day 30 *LINC00662*-CRISPRi neural organoids of PAX6 (green), ZO1 (red), and DAPI (blue). Scale bars = 100 μm. (**F**) DNA methylation profile of the *LINC00662* locus obtained by ONT sequencing of day 15 *LINC00662*-CRISPRi unguided neural organoids compared to control organoids. The top panel shows *LINC00662* in the human genome. The second panel displays ONT read alignments. Unmethylated CpGs colored in the color of the respective sample, and methylated CpGs colored in black. The third panel indicates the CpG position. The fourth panel shows the fraction of methylated CpGs in the region. (**G**) Immunohistochemistry of day 30 *LINC00662*-CRISPRi neural organoids of SOX2 (green), TUJ1 (red), and DAPI (blue). Scale bar, 50 μm. (**H**) Left: Illustration of ROIs for analysis. Right: Quantification of the normalized mean TUJ1 intensities in the different ROIs, two to three rosettes and intermediate zones per organoid from different planes, from six organoids per condition derived from two independent batches from day 15 and day 30 *LINC00662*-CRISPRi organoids. Two-tailed Student’s *t* test. Each datapoint represents the quantified region from an individual organoid slice. n.s., not significant. Created in BioRender. Gerdes, P. (2026) https://BioRender.com/hxodx0i.

To validate the role of *LINC00662* in trans-regulation of proteins related to the cytoskeleton and neurite extension, we generated *LINC00662*-CRISPRi human NPCs (hNPCs) and investigated the subcellular localization of *LINC00662* bound proteins. FLNA, MYH10, and NESTIN all displayed an increased cytoplasmic localization in *LINC00662*-CRISPRi hNPCs when compared to control-transduced cells, indicating that *LINC00662* destabilizes these proteins specifically in the cytoplasm (Fig. 4, C and D).

To investigate whether *LINC00662* is functionally involved in neural differentiation and neurite extension, we generated *LINC00662*-CRISPRi unguided neural organoids. For this experiment, we grew organoids for 15 and 30 days to capture the period when newborn neurons begin to mature and form their first neurites. We found that *LINC00662*-CRISPRi silencing did not affect organoid formation or growth, and the resulting organoids displayed characteristic neural rosettes after 15 days of growth, as visualized by PAX6/ZO1 and SOX2/pH3 staining (Fig. 4E and fig. S3B). The CRISPRi-based silencing of *LINC00662* was also efficient in organoids, and ONT long-read DNA sequencing revealed elevated levels of DNA methylation across the *LINC00662* locus in *LINC00662*-CRISPRi organoids (Fig. 4F). This suggests that the KRAB domain in the CRISPRi system stabilizes the epigenetic silencing of *LINC00662* throughout organoid differentiation, thereby mimicking the epigenetic status of the chimpanzee allele (*62*).

To visualize neuronal differentiation within the organoids, we stained for TUJ1 (beta-III-tubulin), which labels the microtubule cytoskeleton throughout the soma and neurites of newly differentiated neurons, and MAP2, which is enriched in the dendrites of more mature neurons. At 30 days, we observed extensive TUJ1 and MAP2 immunoreactivity together with clear vGLUT1 staining, indicating the presence of differentiated neurons with glutamatergic synaptic structures (Fig. 4G and fig. S3C). However, we noted that the intensity of TUJ1 staining was markedly lower in the *LINC00662*-CRISPRi organoids (Fig. 4G). We quantified TUJ1 staining intensity in the neural rosettes, which are composed of progenitor cells, and the intermediate zone where committed neuroblasts and their neurite extensions are located. As expected, we detected low levels of TUJ1 staining in both the neural rosettes and the intermediate zone on day 15, with no difference between the control and *LINC00662*-CRISPRi organoids (Fig. 4H, two to three rosettes and intermediate zones per organoid from different planes, from six organoids per condition derived from two independent batches from day 15 and day 30). At day 30, we observed a clear increase in TUJ1 labeling intensity in the intermediate zone of control organoids compared to the day 15 time point, confirming the formation of committed neuroblasts with neurite extensions at this time point (Fig. 4H). TUJ1 intensity was significantly reduced in the intermediate zone, but not in the neural rosettes, in *LINC00662*-CRISPRi neural organoids (both gRNAs) (Fig. 4H). We also quantified MAP2 staining intensity, which showed a similar downward trend as TUJ1 in the intermediate zone of *LINC00662*-CRISPRi organoids, although this difference did not reach statistical significance (fig. S3D). Together, these data support a role for *LINC00662* in regulating neurite-associated features during the differentiation of newly committed human neuroblasts.

### Transcriptional alterations in *LINC00662*-CRISPRi neural organoids

Because *LINC00662* is primarily found in the cytoplasm, where it binds to proteins associated with the cytoskeleton and neurite extension, this lncRNA likely regulates neurite extension through a posttranscriptional mechanism. To investigate whether silencing of *LINC00662* has a downstream impact on cell state and transcription we performed snRNA-seq analysis of *LINC00662*-CRISPRi and control neural organoids at 15 and 30 days of differentiation. High-quality data were generated from a total of 119,674 nuclei, including 86,098 from *LINC00662*-CRISPRi organoids (two gRNAs, in total 61 organoids) and 33,576 from control organoids (*lacZ*-gRNA, in total 24 organoids). We performed an unbiased clustering analysis to identify and quantify the different cell types present in the organoids. Seven separate clusters were identified (Fig. 5, A and B), including neural cells of different stages of maturation, such as NPCs and newborn neurons (Fig. 5, C to E). Clear differences in cell type composition were observed between the day 15 and day 30 organoids. To confirm that this separation was not driven by batch effects, we verified that cells from the two batches were well integrated within each condition, whereas the main separation was associated with developmental stage rather than batch (fig. S4A). Day 30 organoids contained more neurons and fewer NPCs, which further demonstrates the presence of more mature cell types in these organoids. We found no apparent difference in the contribution to the different clusters by *LINC00662*-CRISPRi organoids, suggesting that *LINC00662* does not influence developmental fate in unguided neural organoids (Fig. 5, A and B).

**Fig. 5.**
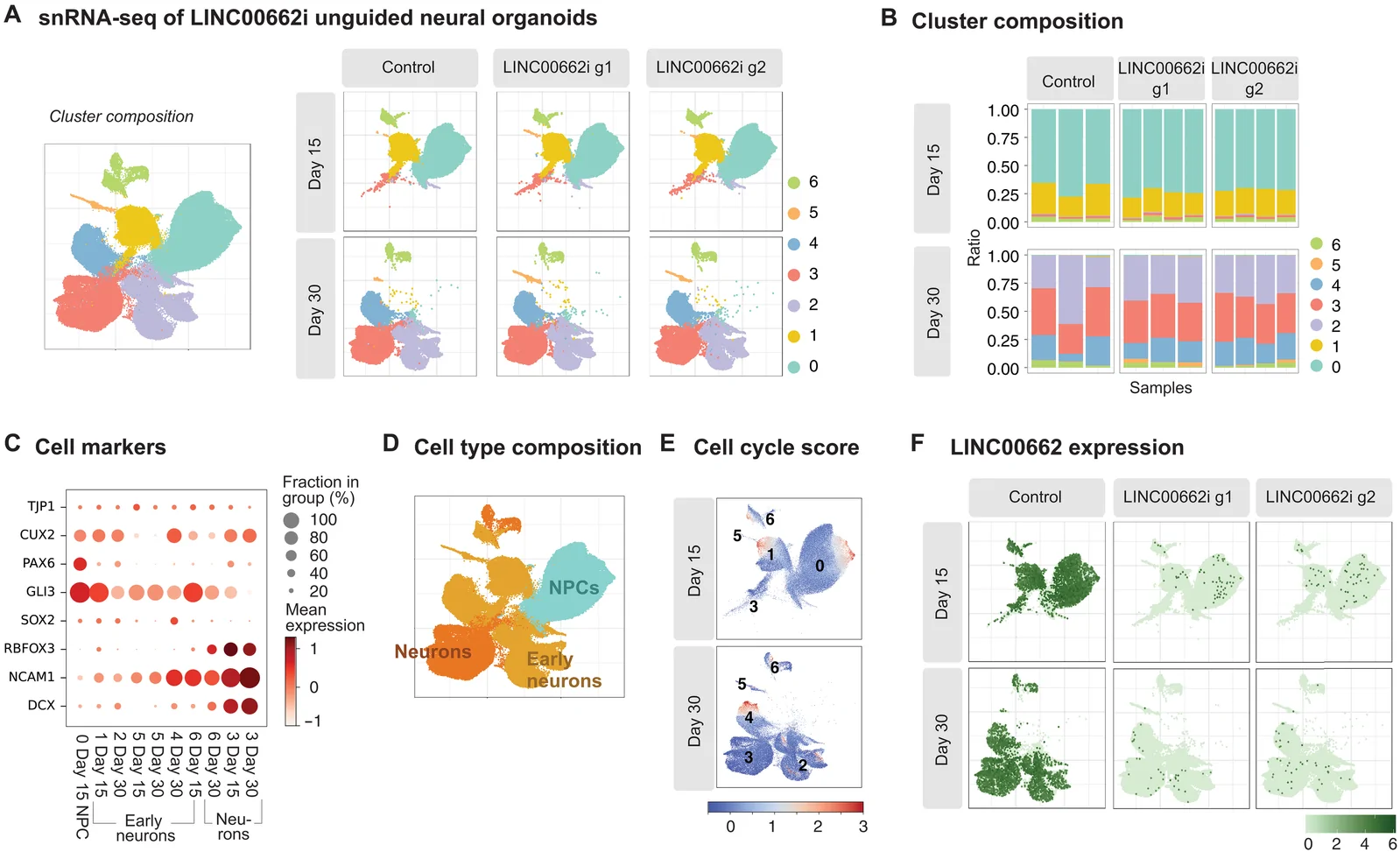
*LINC00662* silencing does not alter neural organoid cell state composition. (**A**) UMAP embeddings of nuclei from day 15 and day 30 unguided neural organoids, showing clustering using all samples (left) and split per condition (right). (**B**) Barplot showing the cluster composition per sample. (**C**) Expression of selected marker genes at both time points (day 15 and day 30). Color indicates the average expression per cluster; dot size indicates the percentage of nuclei in which the gene was detected. (**D**) UMAP colored by annotated cell types. (**E**) UMAP showing cell cycle scores, calculated as the sum of gene module scores for S phase and G_2_-M phase markers (Scanpy:: score_genes_cell_cycle). (**F**) Expression of *LINC00662* in day 15 (top) and day 30 (bottom) of control (left) and *LINC00662*-CRISPRi (right) unguided neural organoids according to snRNA-seq.

Next, we analyzed the transcriptional difference between control and *LINC00662*-CRISPRi organoids. We confirmed the transcriptional silencing of *LINC00662* in all cell populations at both time points (Fig. 5F). To avoid pseudoreplication in our statistics, all downstream analyses were performed on pseudobulks of all nuclei in each sample cluster (summed counts). On day 15, we found very limited transcriptional differences between the *LINC00662*-CRISPRi and control organoids. This suggests that *LINC00662* does not affect the earliest phase of neural differentiation, which is in line with the neurite extension analysis. However, in day 30 *LINC00662*-CRISPRi organoids, we found changes in gene expression of many genes related to neuronal maturation and axon and dendrite formation. Gene set enrichment analyses (GSEAs) of the transcriptional differences identified in *LINC00662*-CRISPRi organoids compared to controls revealed a higher expression of genes related to terms linked to axonogenesis, axon extension, dendrite development, and cellular morphogenesis in three neuronal clusters of day 30 *LINC00662*-CRISPRi organoids (Fig. 6, A to C). In line with this, we found that some of the up-regulated genes in newborn neurons are key players in processes such as neurogenesis, e.g., *NOTCH1* (Notch Receptor-1) (*63*), neuronal connectivity, e.g., *CDH4* (Cadherin-4) (*64*), axon, and dendrite growth, e.g., *SLIT3* (Slit Guidance Ligand 3) (*65*), and *KALRN* (Kalirin RhoGEF Kinase) (*66*, *67*) (Fig. 6, B and C). The observed gene expression changes were very similar for the two different gRNAs. Together, these results demonstrate that CRISPRi-mediated silencing of *LINC00662* leads to transcriptional changes in neural organoids on day 30 but not on day 15 of differentiation. These changes are characterized by increased expression of genes related to axonogenesis and dendrite formation. This confirms that the reduced neurite extension phenotype is due to a posttranscriptional mechanism and suggests that the increased expression of genes linked to this pathway is the consequence of a downstream compensatory mechanism. In summary, these findings further support the role of this lncRNA in controlling human neuronal maturation and neurite extension.

**Fig. 6.**
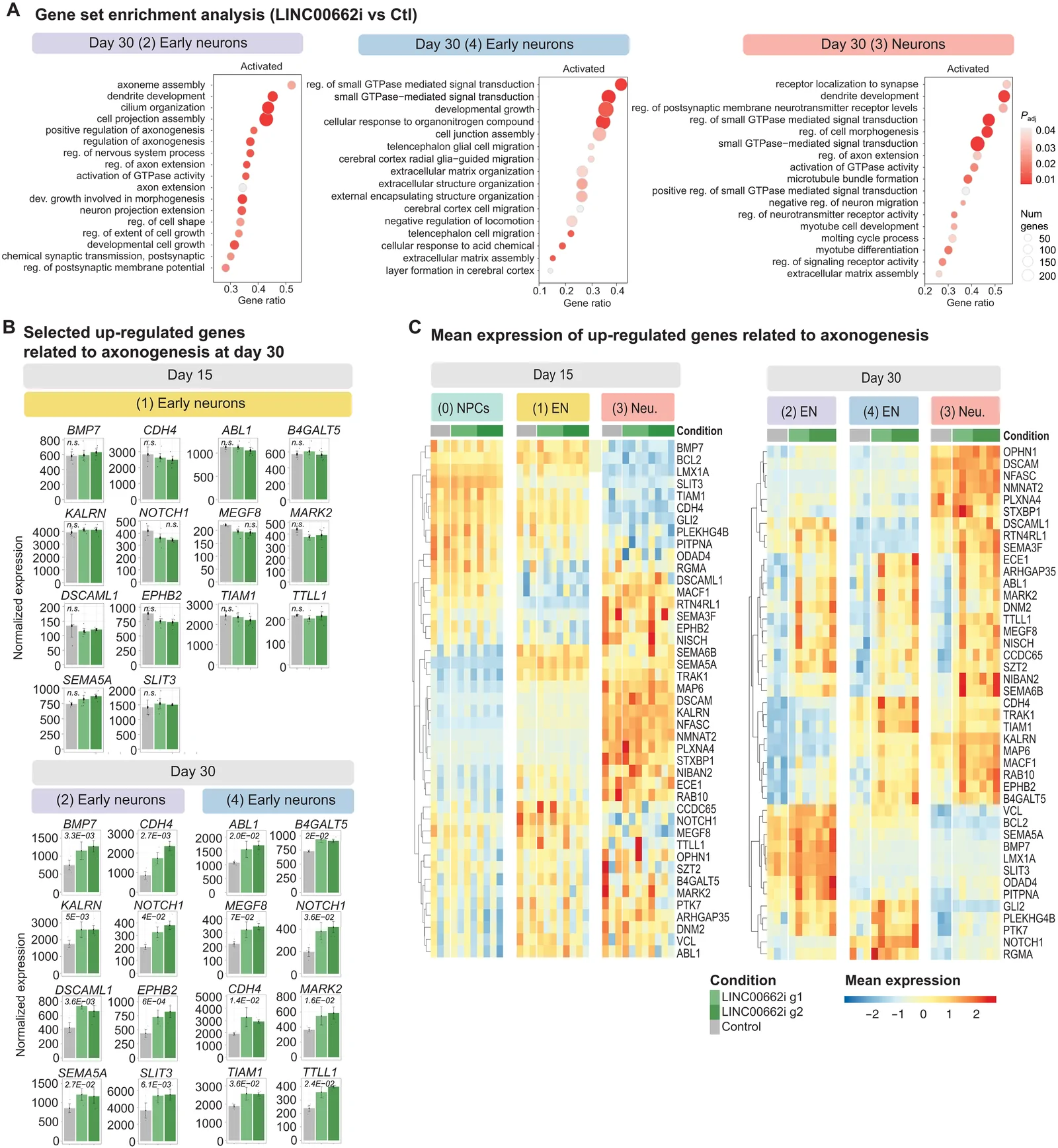
*LINC00662* silencing induces axonogenesis-related transcriptional programs in developing neurons. (**A**) Selected enriched GO terms from the GSEA in early neurons (left and middle) and neurons (right) (clusterProfiler::gseGO). (**B**) Bar plots showing selected up-regulated genes associated with enriched terms [from (A)] (Wald test, DESeq2). Each dot represents the summed expression in the corresponding samples’ cluster (cluster annotated at the top); bars show the group mean with error bars representing the SEM. (**C**) Significantly up-regulated genes among axon-related GO terms [from (A)] in early neurons or neurons (*P*_adj_ < 0.05, Wald test, DESeq2). Color indicates the mean expression in each sample cluster (columns).

## DISCUSSION

Retroviruses are infectious agents that can alter their genetic material and integrate into the host genome as proviruses resulting in the production of new virus copies. In some cases, retroviruses infect germline cells, allowing the integrated proviruses to be passed on to offspring as ERVs (*18*, *32*). PTERV1 likely entered the chimpanzee genome before the separation of humans, chimpanzees, and gorillas, but the human genome was spared the subsequent expansion that occurred in chimpanzees and gorillas between 1.5 and 5 million years ago (*9*). This expansion of PTERV1 in the chimpanzee genome is likely to have had a substantial impact on the population at that time (*9*, *35*, *36*). Endogenization of retroviruses is a relatively infrequent event over evolutionary timescales, making it challenging to observe directly and leaving many aspects poorly understood (*68*). The ongoing endogenization of the koala retrovirus-A (KoRV-A) provides a contemporary parallel to historical events such as the PTERV1 invasion. KoRV-A is currently spreading among wild koala populations via horizontal transmission while simultaneously integrating into germline cells, thereby becoming a component of the inherited genome (*69*, *70*). This process has had a major detrimental impact on the current koala population increasing disease burden, including a high prevalence of cancers (*70*).

Our results support a scenario where PTERV1 infection and endogenization had two effects that influenced hominid speciation. First, it likely drove the ancestor of chimpanzees and bonobos through a population bottleneck (*36*). Second, it resulted in more than 150 fixed PTERV1 insertions in the chimpanzee genome (*9*) (Fig. 1D), some with the potential to affect gene regulatory networks, as exemplified here by *LINC00662*. Thus, our results provide insights into how the colonization of primate genomes by retroviruses influences speciation, including the rewiring of gene regulatory networks during brain development.

Although millions of years have passed since most ERVs were integrated into the primate germ line (*71*, *72*), many ERVs contain sequences that can act as cis-acting regulatory elements (*21*, *22*). As a result, and to protect against their mutagenic potential, genomes have evolved various defense mechanisms against TE invasion (*73*, *74*). KRAB-ZNFs (KZNFs) are the largest family of transcription factors in primate genomes, and their rapid expansion in mammalian genomes correlates with the expansion of TEs (*75*, *76*). Most KZNFs are DNA binding transcriptional repressor proteins that bind to TEs. Their conserved N-terminal KRAB domain interacts with the epigenetic corepressor TRIM28, which induces heterochromatin formation, ultimately leading to the accumulation of DNA methylation that represses the transcriptional and regulatory potential of ERVs and other TEs (*23*, *24*, *26*, *75*–*77*). However, a consequence of this mechanism is the formation of new local mini-heterochromatin domains across species-specific TEs. In this study, we demonstrate that all 158 PTERV1 insertions in the chimpanzee genome are covered by DNA methylation in iPSCs and neural organoids. Although the KZNF responsible for targeting PTERV1 has not yet been identified, our results support a model in which PTERV1s are recognized by the KZNF/TRIM28 repression machinery during early development, resulting in the establishment of DNA methylation across these loci. These local heterochromatin domains are likely to influence gene expression in the vicinity, creating species-specific transcriptional patterns. We identified several transcripts that overlap with a PTERV1 insertion and exhibit differential expression in human and chimpanzee organoids. These include noncoding transcripts such as *LINC00662*, as well as protein coding genes such as for example, the GABA_A_ (γ-aminobutyric acid type A) receptor subunit beta-1 (*GABRB1*), which participates in synaptic transmission and is implicated in the pathogenesis of schizophrenia (*78*, *79*), and the low-density lipoprotein receptor–related protein 1b (*LRP1B*), which has been linked to several malignancies (*80*). Thus, the impact of PTERV1s on the gene regulatory network in chimpanzee cells may be broad and not only limited to **LINC00662*.* In support of this idea, the establishment of heterochromatin domains and spread of DNA methylation into adjacent genomic regions have also been documented for other classes of repetitive elements across diverse species (*6*, *25*, *81*–*84*).

*LINC00662* is an lncRNA that is expressed during human brain development. We found that, in hNPCs, the promoter region of *LINC00662* is hypomethylated and decorated with the active histone mark H3K4me3, consistent with transcriptional activity. In contrast, chimpanzee NPCs exhibit no evidence of *LINC00662* transcription as indicated by the absence of H3K4me3 at the promoter and instead the presence of DNA methylation. This methylated state is likely a consequence of the PTERV1 insertion within intron 1 of *LINC00662* which may facilitate the spread of DNA methylation into the promoter region of *LINC00662*. Notably, LINC00662 expression in chimpanzee cells can be rescued by CRISPR deletion of the PTERV1 element, confirming that the presence of the PTERV1 prevents transcription. In summary, our data suggest that the PTERV1 insertion exerts a repressive influence on the *LINC00662* promoter, but further molecular studies are required to elucidate the precise mechanisms by which the PTERV1 insertion mediates this regulatory effect. Notably, the *LINC00662* locus is absent from the macaque and the gorilla genomes, and a different TE insertion event has occurred in this locus in the orangutan genome, suggesting that only the human genome has a functional *LINC00662* transcriptional unit.

Our data demonstrate that *LINC00662* is a human-specific noncoding transcript, which is expressed during brain development. Although it is challenging to fully characterize the role of a human-specific lncRNA using in vitro organoid modeling, our results support a role for *LINC00662* in the cytoplasm, where it binds to and destabilizes several cytoskeletal proteins involved in neural maturation and axon growth in the developing brain. Consistent with this, our loss-of-function experiments support a posttranscriptional role for LINC00662 in regulating neurite-associated features during the early differentiation of committed neuroblasts. Notably, our snRNA-seq analysis demonstrates that many transcripts related to these processes are up-regulated in immature neurons in *LINC00662*-CRISPRi neural organoids, indicating a downstream feedback loop that activates these transcriptional programs. We do not fully understand the mechanisms underlying this observation, but it could be due to a compensatory mechanism resulting from impaired neurite extension in *LINC00662*-CRISPRi organoids. Our findings provide evidence that posttranscriptional mechanisms may play an important role in the evolution of the human brain. Such mechanisms are challenging to study using transcriptome-based analyses, which currently limits the interpretation of many comparative experiments performed using iPSC-derived neurons or organoids from humans, chimpanzees, and other primate species. Nevertheless, our results provide further support for the idea that differences in dendrite and synapse maturation play a key role in mediating human-specific brain functions (*47*, *85*).

In summary, we demonstrate here how a chimpanzee-specific endogenous retroviral insertion contributes to transcriptional divergence in the development of the human and chimpanzee brains. Our results support the idea that ERVs and other TEs played an important role in the evolution of the hominoid brain.

## MATERIALS AND METHODS

### Identification of full-length PTERV1s and genomic location analysis

The RetroTector software (*38*) (https://github.com/PatricJernLab/RetroTector) was used to mine the chimpanzee genome (version Clint_PTRv2/panTro6 downloaded from https://hgdownload.soe.ucsc.edu/) for ERVs as previously described (*86*). Predicted positions and structures for ERVs scoring 300 and above are summarized in data S1.

To assess the genomic location (intragenic or intergenic) and orientation (sense or antisense) of PTERV1 elements relative to genes, transcript-level annotation was used. The RetroTector output was intersected with the panTro6 RepeatMasker annotation (panTro6.fa.out, downloaded from https://hgdownload.soe.ucsc.edu/) using BEDTools (v2.26.0) to identify PTERV1 elements. Strand information was calculated on the basis of the start and end coordinates of the PTERV1. For transcript-level annotation, the panTro6.ncbiRefSeq.gtf file (downloaded from https://hgdownload.soe.ucsc.edu/) was used. Features annotated as “transcript” were filtered, strand information was retained, and only annotations corresponding to canonical chromosomes 1, 2A, 2B, 3 to 22, X, and Y were included in the analysis.

To identify intragenic PTERV1 elements, BEDTools (v2.26.0) was used using the intersect command with the parameters -a, -b, -wa, and -wb. The resulting overlaps were processed using an awk script to compare the strand orientations of the intersecting features. A new column was added to the output to indicate whether the PTERV1 element and the associated gene were in the “same” or “opposite” orientation. Last, strand orientation frequencies were summarized using an additional awk script to count the occurrences of “same” and “opposite” orientations, avoiding double-counting overlapping PTERV1s.

To identify homologous genes containing PTERV1 insertions in chimpanzee, BEDTools intersect (v2.30.0) was used to compare PanTro6 RefSeq gene annotations (-a) and RetroTector-predicted PTERV1 annotation (-b). Coordinates of genes hosting an entire PTERV1 insertion (-wa -F 1) without an annotated homolog in the human reference genome version hg38 were lifted to hg38 coordinates using the UCSC LiftOver web browser tool.

Last, the resulting lifted coordinates (-a) were intersected with human GENCODE v38 gene annotation (-b) using BEDTools intersect with default parameters (-wo). This analysis identified 45 genes annotated in hg38 with homology to pantro6 genes containing PTERV1 insertions. These candidates were further scrutinized on the basis of their difference of expression between human and chimpanzee organoids using bulk RNA-seq (see “Bulk RNA-seq analysis of human and chimpanzee organoid samples”).

### iPSC culture

The two chimpanzee iPSC lines used in this study have been generated in other labs and previously reported: Sandra A (female) (*41*), generated with nonintegrative episomal vectors, here referred to as PT1, and Jo_C (male) (*40*), generated with nonintegrative episomal vectors, here referred to as PT2. The two human iPSC lines used in this study were obtained from RIKEN. These lines were generated using nonintegrative episomal vectors (*87*): RBRC-HPS0328 606A1 (female), here referred to as HS1 (RIKEN, RRID:CVCL_DQ11), and RBRC-HPS0331 610B1 (male), here referred to as HS2 (RIKEN, RRID: CVCL_DQ4).

The iPSC lines were maintained on LN521 (0.7 μg/cm^2^; Biolamina) coated Nunc multidishes in iPS media (StemMACS iPS-Brew XF and 0.5% penicillin/streptomycin; GIBCO). iPSCs were passaged 1:2 to 1:6 every 2 to 3 days when 70 to 90% confluency was reached. Media change was performed daily, and 10 μM Y27632 (Rock inhibitor, Miltenyi) was added when cells were passaged.

### hNPC culture

The embryo-derived hNPC line (Sai2) is derived from fetal brain tissue at Carnegie stage 16 (*88*). These hNPCs have the characteristics of neuroepithelial-like stem cells and were maintained according to an established protocol (*89*). The hNPCs were cultured in poly-l-ornithine (15 μg/ml; Sigma-Aldrich) and Laminin-2020 (2 μg/ml; Sigma-Aldrich) coated wells in culture media Dulbecco’s modified Eagle’s medium (DMEM)/F-12 (Thermo Fisher Scientific), N2 supplement (1X, Thermo Fisher Scientific), l-glutamine (2 mM; Sigma-Aldrich), penicillin/streptomycin (1×, Gibco), B27 (0.05×, Thermo Fisher Scientific), epidermal growth factor (EGF) (10 ng/ml; Thermo Fisher Scientific), and fibroblast growth factor (FGF) (10 ng/ml; Sigma-Aldrich) with daily media change. The hNPCs were passaged every 2 to 3 days using TrypLe express (Gibco).

### Unguided neural organoid culture

Human and chimpanzee unguided neural organoids were generated using the STEMdiff Cerebral Organoid kit (STEMCELL Technologies) following the manufacturer’s instructions with some adjustments. Briefly, human (HS1 and HS2) and chimpanzee (PT1 and PT2) iPSCs were maintained in StemMACS iPS brew XF as described under “iPSC culture.” Chimpanzee iPSCs were transferred to mTeSR1 medium (STEMCELL Technologies) 2 weeks before starting the organoids and maintained as described under “iPSC culture” with mTeSR1 replacing StemMACS iPS brew XF. After that, the timing of the STEMdiff Cerebral Organoid kit protocol was followed with the exception of PT2, which was transferred to neural induction medium 1 day earlier than described. In addition, EBs for HS1, HS2, and PT1 were generated using the kit’s EB seeding medium but EBs for PT2 using mTeSR1. Briefly, at day 0 of differentiation, iPSCs at 70 to 90% confluency were detached and centrifuged and then resuspended in 1 ml of EB Seeding Medium [STEMdiff Cerebral Organoid Basal Medium 1 and STEMdiff Cerebral Organoid Supplement A, 4:1, supplemented with 10 μM of Y-27632 (Rock Inhibitor)] or mTeSR1 [supplemented with 10 μM Y-27632 (Rock Inhibitor)]. A total of 9000 cells per well in 100 μl of EB seeding medium (HS1, HS2, and PT1) or 10,000 cells in 250 μl of mTeSR1 [supplemented with 10 μM Y-27632 (Rock Inhibitor); PT2] were then distributed in a round-bottom ultralow attachment 96-well plate. EBs were incubated at 37°C for 48 hours. At days 2 and 4 of differentiation, 100 μl of fresh EB Formation Medium (STEMdiff Cerebral Organoid Basal Medium 1 and STEMdiff Cerebral Organoid Supplement A, 4:1; HS1, HS2, and PT1) or 200 μl of mTeSR1 (PT2) was added to each well. On day 5, or when the organoids reached a diameter of >300 μm, organoids were transferred to an ultralow attachment 24-well plate with 0.5 ml of Induction Medium (STEMdiff Cerebral Organoid Basal Medium 1 and STEMdiff Cerebral Organoid Supplement B) in each well (one organoid per well to avoid organoid fusion). Organoids were incubated at 37°C for 48 hours. On day 7, organoids were embedded with ∼15 μl of Matrigel per organoid on an embedding surface (Parafilm) in a sterile 100-mm dish and incubated at 37°C for 30 min to polymerize the Matrigel. Organoids were then transferred to an ultralow attachment 6-well plate with 3 ml of Expansion Medium (STEMdiff Cerebral Organoid Basal Medium 2, STEMdiff Cerebral Organoid Supplement C, and STEMdiff Cerebral Organoid Supplement D) per well. Ten to 12 organoids per well were cultured together. On day 10, the organoids were transferred to an orbital shaker {rpm = 1000 × √[*RCF*/(1.118 × d)]}, and media were changed with 3 ml per well of Maturation Medium (STEMdiff Cerebral Organoid Basal Medium 2 and STEMdiff Cerebral Organoid Supplement E). The medium was replaced every 3 days. Two independent batches of organoids per cell line were generated. On day 15 of differentiation, the organoids were collected for downstream analyses. Five organoids were collected and snap frozen for each bulk RNA-seq replicate (*n* = 3 replicates per cell line per batch). Five organoids were collected and snap frozen for each snRNA-seq replicate (*n* = 1 replicate per cell line per batch). Three organoids were collected for immunostaining (see “Immunohistochemistry”). Five organoids were collected for long-read DNA sequencing.

### Immunocytochemistry

The iPSCs and hNPCs were washed two times with KPBS for 5 min and subsequently fixed with 4% paraformaldehyde for 15 min at room temperature. This was followed by two washes with KPBS for 5 min. The cells were blocked and permeabilized in blocking solution (0.3% Triton X-100 and 5% normal donkey serum in KPBS) for 45 min at room temperature. Primary antibodies [rabbit anti-NANOG (Abcam, AB446437), 1:1000 dilution; mouse FLNA (Proteintech, 67133-1-Ig), 1:400 dilution; rabbit anti-MYH10 (Proteintech, 19673-1-AP), 1:200 dilution, rabbit anti-NESTIN (Abcam, AB176571), 1:100 dilution; and chicken anti-MAP2 (Abcam, AB5392), 1:1000 dilution] were added in blocking solution and incubated overnight. On the next day, the cells were washed two times with KPBS for 5 min. The secondary antibody [donkey anti-rabbit Alexa Fluor 488 (Invitrogen, A21206), 1:500; donkey anti-rabbit Cy5 (Jackson ImmunoResearch, AB_2340607), 1:500; donkey anti-mouse Cy3 (Jackson ImmunoResearch, 715-166-075), 1:500 dilution; donkey anti-rabbit Cy3 (Jackson ImmunoResearch, 711-165-152), 1:500 dilution; or donkey anti-chicken Alexa Flour 488 (Jackson ImmunoResearch, 703-546-155), 1:500 dilution] was added in blocking solution and incubated at room temperature for 1 hour followed by three washes with KPBS for 5 min. 4′,6-Diamidino-2-phenylindole (DAPI) (1:1000; Sigma-Aldrich) in KPBS was added as a nuclear counterstain and incubated for 5 min at room temperature. The cells were washed two times with KPBS for 5 min and imaged on a Leica DMI6000B fluorescence microscope.

Quantification of immunofluorescence staining was performed using ImageJ Fiji. Nuclear and cell body masks were defined using DAPI-positive and MAP2-positive staining, respectively, to generate regions of interest (ROIs). Cytoplasmic ROIs were defined by subtracting the nuclear ROI from the corresponding cell body ROI. The defined nuclear and cytoplasmic ROIs were applied to the original FLNA, MYH10, or NESTIN images, and mean fluorescence intensities were measured per cell. Nuclear to cytoplasmic ratios were calculated by subtracting the cytoplasmic mean fluorescence intensity from mean fluorescence intensity in the corresponding nucleus.

For each protein of interest, three wells with *lacZ* hNPCs and three wells with *LINC00662*-CRISPRi g2 hNPCs were analyzed. One to two images were analyzed per well, and 8 to 10 cells were quantified per image resulting in a total of 46 to 60 cells analyzed per condition. Statistical analysis was performed in GraphPad Prism using an unpaired *t* test with Welch’s correction (GraphPad Software, San Diego, USA). Figures were assembled in ImageJ (NIH) and Adobe Photoshop (Adobe Systems, San Jose, CA); only contrast and brightness were adjusted to optimize the image quality.

### Immunohistochemistry

Unguided neural organoids were fixed in 4% paraformaldehyde for 1 hour at room temperature. After that, they were washed three times with KPBS and left in a 1:1 30% sucrose solution and OCT (catalog no. 45830, HistoLab) mixture overnight at 4°C. Organoids were then transferred to a cryomold containing OCT, frozen on dry ice, and stored at −80°C in freezer bags.

Ahead of staining, organoids were sectioned on a cryostat at −20°C at a thickness of 20 μm and placed onto Superfrost plus microscope slides. They were then washed two times with KPBS for 5 min and subsequently blocked and permeabilized in 0.25% Triton X-100 and 4% normal donkey serum in KPBS for 1 hour at room temperature. The primary antibodies [rabbit anti-PAX6 (BioLegend, 901301), 1:500 dilution; mouse anti-ZO1 (Invitrogen, 339100), 1:300 dilution; rabbit anti-beta tubulin III (BioLegend, 802001), 1:1000 dilution; goat anti-SOX2 (R&D Systems, AF2018), 1:100 dilution; rabbit anti-phospho-Histone H3 (Ser^10^) (Cell Signaling Technologies, 3377), 1:600 dilution; chicken anti-MAP2 (Abcam, 5392), 1:1000 dilution; and rabbit anti-VGLUT1 (Synaptic Systems, SYSY135302), 1:1000 dilution] were prepared in antibody solution (0.1% Triton X-100 and 4% normal donkey serum in KPBS), added to the sections and incubated overnight at 4°C. Subsequently, the sections were washed two times with KPBS and one time with wash solution (0.5% Triton X-100 in KPBS). The secondary antibody [donkey anti-rabbit Alexa Fluor 488 (Invitrogen A21206), 1:1000; donkey anti-rabbit Cy3 (Jackson ImmunoResearch AB_2307443), 1:400; donkey anti-goat Alexa Fluor 488 (Invitrogen A11055), 1:500; donkey anti-rabbit Alexa Fluor 568 (Invitrogen A10042), 1:500; and donkey anti-mouse Alexa Fluor 647 (Invitrogen A31571), 1:1000] was prepared in antibody solution, added to the sections, and incubated at room temperature for 1 hour protected from light, followed by two washes with KPBS and one wash with washing solution. The sections were incubated with DAPI in KPBS (1:1000) for 5 min at room temperature and washed once with KPBS. The sections were mounted using FluorSave (Merck, 345789) and imaged using an Operetta CLS (PerkinElmer) or on a Leica DMI6000B fluorescence microscope. Figures were assembled in ImageJ (NIH) and Adobe Photoshop (Adobe Systems, San Jose, CA); only contrast and brightness were adjusted to optimize the image quality.

Mean fluorescence intensities of DAPI and TUJ1/MAP2 were measured for two to three ROIs (rosette and intermediate zone) from six organoids derived from two independent batches at 15 and 30 days. Regions were chosen manually in a blinded manner based on DAPI staining after (i) manually marking the rosette ventricle followed by automatic expansion of the region with 18 pixels leaving a donut-shaped ROI and (ii) manually drawing intermediate zone following the rosette edge using ImageJ (NIH). To control for variation in cell number across sections, TUJ1/MAP2 values were normalized to the corresponding mean DAPI fluorescence intensity for each ROI. Two-tailed Student’s *t* test or one-way analysis of variance (ANOVA) followed by Dunnett’s test using α = 0.5 was conducted. Statistical analysis was performed in GraphPad Prism (GraphPad Software, San Diego, USA).

### Single-nucleus RNA-seq

Nuclei isolation from organoids was performed as described previously (*90*). Briefly, the organoids were thawed and dissociated in ice-cold lysis buffer [0.32 M sucrose, 5 mM CaCl_2_, 3 mM MgAc, 0.1 mM Na_2_EDTA, 10 mM tris-HCl (pH 8.0), and 1 mM dithiothreitol (DTT)] using a 1-ml tissue douncer (Wheaton). The homogenate was carefully layered on top of a sucrose solution [1.8 M sucrose, 3 mM MgAc, 10 mM tris-HCl (pH 8.0), and 1 mM DTT] and centrifuged at 30,000*g* for 2 hours and 15 min. Pelleted nuclei were softened for 10 min in 100 μl of nuclear storage buffer [15% sucrose, 10 mM tris-HCl (pH 7.2), 70 mM KCl, and 2 mM MgCl_2_], resuspended in 300 μl of dilution buffer [10 mM tris-HCl (pH 7.2), 70 mM KCl, and 2 mM MgCl_2_], and ran through a cell strainer (70 μm). The nuclei were sorted via FANS (with a FACSAria, BD Biosciences) at 4°C at low flow rate using a 100-μm nozzle (reanalysis showed >95% purity).

Fluorescence-activated cell sorting (FACS)–sorted nuclei (8500 to 10,000 nuclei/cells per sample) were directly loaded onto the Chromium Next GEM Chip G Single Cell Kit along with the reverse transcription mastermix following the manufacturer’s protocol for the Chromium Next GEM single cell 3′ kit (PN-1000268, 10X Genomics) to generate single-cell gel beads in emulsion. cDNA amplification was done according to the guidelines from 10X Genomics using 13 cycles of amplification for 3′ libraries. Sequencing libraries were generated with unique dual indices (TT set A) and pooled for sequencing on a NovaSeq 6000 or NovaSeq X Plus using a 100-cycle kit and 28-10-10-90 reads.

#### snRNA-seq analysis

Basecalling of reads and generation of sample-specific FASTQ files were performed using 10X Genomics mkfastq (v6.0.0; RRID:SCR_017344). Alignment to the reference genome (hg38) and gene quantification was performed using 10X Genomics Cell Ranger count (v6.0.0) including intronic reads (--include-introns).

#### snRNA-seq analysis of human and chimpanzee organoids

*Quality control.* Preprocessing and quality control of all samples were conducted using Seurat (v5.1.0). Read count matrices (produced by Cell Ranger) were loaded into individual Seurat objects using Seurat::Read10X. Genes expressed in fewer than three nuclei in the whole dataset were excluded when creating the individual Seurat objects (Seurat::CreateSeuratObject, min.cells = 3). Nuclei containing more than 2% mitochondrial gene counts, with less than 500 genes detected, or with a number of genes detected over 2 SDs, or under 1 SD from the sample’s mean, were excluded from further analyses.

*Clustering and UMAP embeddings.* Samples’ objects were integrated using Seurat::FindIntegrationAnchors (dims = 1:30) and Seurat::IntegrateData (dims = 1:30). Count scaling was performed using Seurat::ScaleData across all genes. Principal components analysis (PCA) was conducted using Seurat::RunPCA using the most variable features on the dataset (Seurat::VariableFeatures). Neighborhood graph construction was based on 10 PCAs and 20 nearest neighbors (Seurat::FindNeighbors, dims = 1:10, k.param = 20). Clustering was performed using Seurat::FindClusters with a resolution of 0.1 (res). Uniform Manifold Approximation and Projection (UMAP) reduction was calculated using Seurat::RunUMAP (dims = 1:10).

*Cell type annotation.* Cell cycle scores were calculated using a previously established list of cell cycle–related genes using Seurat::CellCycleScoring. Cell type annotations were manually assigned on the basis of the expression of canonical gene markers.

#### snRNA-seq analysis of human LINC00662-CRISPRi organoids

*Quality control.* Preprocessing and quality control of all samples were conducted using Scanpy (v1.10.1). Read count matrices (produced by Cell Ranger) were loaded into individual AnnData objects using Scanpy::read_10x_mtx. Genes expressed in fewer than three nuclei in the whole dataset were excluded using Scanpy::filter_genes (min_cells = 3).

Quality control metrics were computed with Scanpy::calculate_qc_metrics, considering mitochondrial and ribosomal gene content (percent_top = None, log1p = False). Doublets identified by Scanpy::Scrublet were excluded from further analyses. Nuclei containing more than 2% mitochondrial gene counts, with less than 500 genes detected, or with a number of genes detected outside 2 SDs from the sample’s mean, were excluded from further analyses.

*Clustering and UMAP embeddings.* Samples were merged using Anndata::concat (join = “outer”). Count normalization was performed with Scanpy::pp.normalize_total (target sum = 1 × 10^6^), followed by natural log transformation (Scanpy::pp.log1p) and scaling (Scanpy::pp.scale, max_value = 10).

PCA was conducted using the arpack solver (Scanpy::tl.pca, svd_solver = “arpack”). Neighborhood graph construction was based on 20 PCAs and 15 nearest neighbors (Scanpy::pp.neighbors, n_neighbors = 10, n_pcs = 20). Clustering was performed using the Leiden algorithm (Scanpy::tl.leiden, resolution = 0.1, directed = False, flavor = “igraph”).

A graph abstraction was generated via partition-based graph abstraction (PAGA) (Scanpy::tl.paga), and the UMAP layout was computed using PAGA-initialized positions (Scanpy::tl.umap, init_pos = “paga”). Upon examination of UMAP embeddings, batch effects (e.g., experimental organoid batches) were integrated using Harmony (Scanpy::external.pp.harmony_integrate). The nearest-neighbor graph was then recomputed using the Harmony-adjusted PCA (Scanpy::pp.neighbors, use_rep = “X_pca_harmony”), followed by reclustering with Leiden and recomputation of PAGA and UMAP using the same parameters as above.

*Cell type annotation.* Cell cycle scores were calculated using a previously established list of cell cycle–related genes using Scanpy::tl.score_genes_cell_cycle. Cell type annotations were manually assigned on the basis of the expression of canonical gene markers.

*Pseudobulks and gene differential expression analyses.* Raw gene counts were summed per cluster per sample, to produce seven (clusters 0 to 6 as shown in Fig. 5D) cluster-specific gene count matrices where each column is a sample, and each gene is a row. These pseudobulked gene count matrices were input to DESeq2 with a pseudocount of 1 to perform differential expression analyses between *LINC00662*-CRISPRi and control organoids per time point (day 15 and day 30) per cluster (clusters 0 to 6). Design for the test was set to ∼ batch + condition to test for transcriptional differences between conditions (*LINC00662*-CRISPRi/Control) while accounting for experimental organoid batches. Log_2_FoldChanges were shrunk using DESeq2::lfcShrink.

Gene log_2_FoldChanges were ranked and input as a named list to clusterProfiler::gseGO from which a minimum gene set size was set to 3 (minGSSize) and a maximum of 800 (maxGSSize). Bonferroni and Hochberg correction (pAdjustMethod = “BH”) was performed on the results, and a *P* value cutoff of 0.05 (pvalueCutoff) was used to filter significant terms. Heatmap visualization (Fig. 6C) was performed on mean normalized counts (DESeq2::counts, normalized = T) using pheatmap scaling per gene (scale = “row”).

### Bulk RNA-seq

Total RNA was isolated from organoids using the RNeasy Mini Kit (QIAGEN). Libraries were generated using Illumina TruSeq Stranded mRNA library prep kit [poly(A) selection] and sequenced on a NovaSeq 6000 or NovaSeq X Plus (PE, 2 × 150 bp).

#### Bulk RNA-seq analysis of human and chimpanzee organoid samples

Reads were aligned to the reference genome (hg38) using STAR aligner (v2.7.8a) using a unique mapping approach (--outFilterMultimapNmax 1) with a maximum allowed mismatch of 3% (--outFilterMismatchNoverLmax 0.03). Alignment files were sorted and indexed using SAMtools (v1.16.1) and input to deeptools BAMCoverage function (v2.5.4) splitting strands (--filterRNAstrand) and normalizing using RPKM (--normalizeUsing RPKM).

*Gene differential expression analysis.* Gene count matrices of Gencode v38 were produced using featureCounts (Subread package, v1.6.3, -s 2). Differential expression analysis between the two species was performed using DESeq2 (v1.44.0, design = ∼species). Log_2_FoldChanges were shrunk using DESeq2::lfcShrink. Heatmap visualization (Fig. 2B) was performed on mean normalized counts (DESeq2::counts, normalized = T) using pheatmap (scale = “none”).

### Long-read DNA sequencing

High-molecular-weight (HMW) DNA was extracted from frozen human fetal brain tissue and human and chimpanzee iPSC pellets (500,000 to 1 million cells) and day 15 unguided neural organoids (five organoids) using the Nanobind HMW DNA Extraction kit (PacBio) following the manufacturer’s instructions. HMW DNA was eluted in 100 μl of Buffer EB. Size selection to enrich for fragments >5 kb was performed using the Short Read Eliminator XS kit (PacBio) according to the manufacturer’s instructions. HMW DNA quantity and quality were assessed by NanoDrop and Qubit from the top, middle, and bottom of each tube and on a TapeStation 4200 (Agilent) using the Agilent Genomic DNA Screen Tape Assay (ref. no. G2991-90040, Edition 08/2015). HMW DNA was sheared to ∼10-kb fragment length using a g-TUBE (Covaris) following the manufacturer’s protocol. Library preparation for whole-genome sequencing was performed using the SQK-LSK114 Ligation Sequencing kit (ONT) with >1 mg of DNA input. Libraries were each sequenced separately using a FLO-PRO114M PromethION Flow Cell R10.4.1 on a PromethION platform (ONT) at SciLifeLab Uppsala or in-house on a PromethION 2 Solo (ONT) for 72 hours.

#### Long-read DNA analysis

Raw sequencing data (POD5 format) were basecalled and mapped to either the human reference genome (GRCh38/hg38) or chimpanzee reference genome (Clint_PTRv2/panTro6) using Dorado version 0.5.1-CUDA-11.7.0 (https://github.com/nanoporetech/dorado) using the super accurate basecalling model dna_r10.4.1_e8.2_400bps_sup@v4.3.0 and dna_r10.4.1_e8.2_400bps_sup@v4.3.0_5mCG_5hmCG@v1 for methylation aware basecalling. Resulting BAM files were sorted and indexed using SAMtools version 1.18 (*91*). PTERV1 and *LINC00662* methylation were visualized with MethylArtist version 1.2.4 (*92*) using the segmeth (parameters:–excl_ambig), segplot, and locus functions with default parameters.

### Long-read direct RNA-seq

#### RNA extraction

RNA was extracted from fetal forebrain samples using the TRIzol RNA extraction protocol (catalog no. 15596026). Briefly, the tissue was homogenized in 1 ml of TRIzol reagent (Invitrogen; pub. no. MAN0001271). Two hundred microliters of chloroform was then added and mixed to the lysate after 5 min of incubation at room temperature. After 3 min of incubation (room temperature), the sample was centrifugated at 12,000*g* at 4°C for 20 min. The aqueous phase (containing the RNA) was transferred to a new tube, and 500 μl of isopropanol was added, followed by a 10-min incubation at 4°C. RNA was then pelleted by centrifugation at 12,000*g* for 10 min at 4°C. RNA was resuspended in 1 ml of 75% ethanol, vortexed, and centrifuged for 5 min at 7500*g* at 4°C. The RNA pellet was air dried and resuspended in 35 μl of RNase-free water. The suspension was then incubated at 56°C for 15 min. DNase treatment (pub. no. 1907 M) was then performed on the isolated RNA using the TURBO DNA-free Kit (Invitrogen, catalog no. AM1907) according to the manufacturer’s instructions. Poly(A) mRNA isolation, library preparation, and sequencing were performed as previously described (*93*).

#### Long-read direct RNA analysis

Basecalling was performed using dorado (version 0.7.1-CUDA-11.7.0) with model rna004_130bps_sup@v3.0.1 and modified bases model rna004_130bps_sup@v3.0.1_m6A_GRACH@v1 (--modified-bases-models). Reads were mapped to the reference genome (hg38) using minimap2 (-ax splice -uf -k14 -y). Genome browser visualization was done in IgV (version 2.18.2) (http://broadinstitute.org/igv/) using the sorted and indexed alignment files (samtools 1.16.1).

### Generation of *LINC00662-*PTERV1 knockout chimpanzee iPSCs

#### Lentiviral production

Lentivirus was produced as previously described (*94*). Human embryonic kidney (HEK) 293T cells (RRID:CVCL_0063) at a confluency of 70 to 90% were transfected with third-generation packaging and envelope vectors pMDL (Addgene, #12251), psRev (Addgene, #12253), and pMD2G (Addgene, #12259) with polyethyleneimine (PEI Polysciences, PN 23966) in Dulbecco’s phosphate-buffered saline (DPBS) (GIBCO). The lentivirus was harvested 48 hours after transfection by filtering and centrifugation at 25,000*g* for 1.5 hours at 4°C. The virus was resuspended in DPBS, aliquoted, and stored in −80°C until further use. Lentiviruses were in titers of 10^9^, determined by standard quantitative RT-PCR (qRT-PCR).

#### CRISPR-Cas9 gene editing

The chimpanzee cell line PT1 was dissociated using Accutase, and 200,000 cells were plated in laminin-521–coated wells in iPS media with 10 μM Y27632. Cells were transduced with lentiviral vectors pLV.U6.EFS-NS.H2B-RFPW (Addgene, plasmid #170363; RRID:Addgene_170363) with a multiplicity of infection (MOI) of 1 and pLV.CAS9-GFP (Addgene, plasmid #170361; RRID:Addgene_170361) with an MOI of 20 carrying sgRNA1 and sgRNA2, respectively, designed to target the flanking regions of PTERV1. Cells were expanded for 10 days before FACS (FACSAria III, BD Biosciences). Cells were detached using Accutase and resuspended in iPS media containing Y27632 (10 μM) and DRAQ7 (1:1000). Gating parameters were determined by side and forward scatter to eliminate debris and aggregated cells. The green fluorescent protein–positive (GFP^+^) and red fluorescent protein–positive (RFP^+^) gates were set using untransduced and single transduced iPSCs. The sorting gates and strategies were validated via reanalysis of sorted cells (>99% purity cutoff). A total of 300,000 RFP^+^/GFP^+^/DRAQ7^−^ cells were collected, spun down at 400*g* for 5 min, and kept at −80°C until further analysis.

sgRNA1: GCAGCACTGCGCATCTCTGA

sgRNA2: ACCAGGTAATGAAAAGAGCC

To confirm successful excision of PTERV1, genomic DNA was extracted from wild-type (WT) and PTERV1-excised PT1 cells according to the manufacturer’s instructions (DNeasy Blood and Tissue kit, Qiagen). Using genomic primers targeting *LINC00662*, a 7500-bp region was amplified (PrimeSTAR GXL DNA Polymerase, TAKARA). The PCR product of the expected size was purified (QIAquick Gel Extraction Kit, Qiagen) and confirmed through automated Sanger sequencing (EuroFins genomics).

gPCR_*LINC00662*_Fwd: GCGGCTGATCTCACCTTGTA

gPCR_*LINC00662*_Rev: ACGCAGCAGGACAGAATCTC

### Quantitative RT-PCR

Total RNA isolated from iPSCs or unguided neural organoids (RNeasy mini kit, Qiagen) was reverse transcribed using the Maxima cDNA synthesis kit (Thermo Fisher Scientific). qRT-PCR was performed using primers listed below and SYBR Green Master Mix (Roche) on a LightCycler 480. Three technical replicates per sample were amplified for 40 cycles, and cycle values were normalized to β-*actin* and *HPRT*.

Primer sequence (5′ to 3′)

*LINC00662* Fwd: CAGGCCACTTACAGACTCCA

*LINC00662* Rev: AGTGTTTCCAGCCTGAGACT

β-*actin* Fwd: CCTTGCACATGCCGGAG

β-*actin* Rev: GCACAGAGCCTCGCCTT

*HPRT* Fwd: ACCCTTTCCAAATCCTCAGC

*HPRT* Rev: GTTATGGCGACCCGCAG

### fbNPC differentiation

iPSC differentiation into forebrain neural progenitors (fbNPCs) was performed as previously described (*6*, *95*). Briefly, iPSCs at 70 to 90% confluency were dissociated using Accutase and plated in N2 differentiation medium [1:1 DMEM/F-12 (21331020; GIBCO) and Neurobasal (21103049; GIBCO) supplemented with 1% N2 (GIBCO), 2 mM l-glutamine (GIBCO), and 0.2% penicillin/streptomycin] on LN111-coated (1.14 μg/cm^2^; Biolamina) Nunc Δ multidishes at a density of 10,000 cells/cm^2^ with 10 μM SB431542 (Axon) and noggin (100 ng/ml; Miltenyi) for dual SMAD inhibition, and 10 μM Y27632. Media (N2 medium + SB + noggin) was changed every 2 to 3 days. On day 9 of differentiation, media were changed to N2 media without SMAD inhibitors. On day 11, cells were replated in B27 medium [Neurobasal supplemented with 1% B27 without vitamin A (GIBCO), 2 mM l-glutamine, 0.2% penicillin/streptomycin, brain-derived neurotrophic factor (BDNF) (20 ng/ml; R&D), l-ascorbic acid (0.2 mM; Sigma-Aldrich), and Y27632 (10 μM)] on LN111-coated Nunc Δ multidishes at a density of 800,000 cells/cm^2^. Cells were harvested at day 14 for downstream analysis.

### Cellular fractionation assay

Cell fractionation was performed using the Paris kit (Thermo Fisher Scientific; part no. AM1921) on HS1 iPSC-derived fbNPCs. Briefly, nuclear and cytoplasmic fractions were separated from fresh cultured cells. In parallel, total RNA was extracted from whole-cell lysate from the same culture. RNA was isolated from whole cells and nuclear and cytoplasmic lysate individually according to the manufacturer’s instructions. Isolated RNA was reversed transcribed into cDNA and analyzed using qRT-PCR as described above.

### ChOP mass spectrometry

This experimental workflow was based on a ChIRP-MS protocol published earlier (*96*). HS1 iPSCs were used to generate fbNPCs as described above. On day 14, fbNPCs were trypsinized, washed with DPBS, and spun down at 500*g* for 5 min. Cells were resuspended in 1% formaldehyde in DPBS and kept at 1 × 10^6^ to 2.5 × 10^6^ cell/ml of 1% formaldehyde. Cells were incubated at room temperature for 30 min with end-to-end rotation. The reaction was stopped with 0.125 mM glycine for 5 min with end-to-end rotation. Cells were pelleted at 4000*g* for 5 min before being rinsed with DPBS and pelleted again. The supernatant was discarded, and the pellet was processed for DNase digestion.

To digest genomic DNA, pellets were treated with 200 μl of TURBO nuclear lysis buffer [50 mM Tris (pH 7.0), 0.5% NP-40, 1× Turbo DNase buffer, 1× protease inhibitor cocktail, 1 mM phenylmethylsulfonyl fluoride (PMSF), TURBO DNase (10 U/ml), and RNase inhibitor (0.1 U/μl)], followed by the addition of 10 μl of enzyme per sample. Incubation was carried out at 37°C for 30 min. Reactions were stopped by adding 850 μl of STOP lysis buffer [50 mM Tris (pH 7.0), 50 mM EDTA, 50 mM EGTA, 1% SDS, 0.5% NP-40, 1 mM DTT, 1 mM PMSF, protease inhibitor, and RNase inhibitor (0.1 U/μl)], followed by 15-min incubation on ice with intermittent pipetting. Nuclear homogenization was achieved by passing the lysate through a syringe needle.

Sonication was performed using Bioruptor with 30 s ON/OFF cycles for a total of 30 cycles, or until the solution became clear. The chromatin was clarified by centrifugation (16,000*g*, 10 min, 4°C), and the supernatant was pooled across replicates. Hybridization buffer [50 mM Tris (pH 7.0), 10 mM EDTA, 1% SDS, 1 mM DTT, 750 mM NaCl, 15% formamide, 1 mM PMSF, protease inhibitors, and RNase inhibitor (0.1 U/μl)] was added in a 2:1 ratio to the chromatin volume.

For preclearing, 100 μl of C1 beads per 100 million cells were washed twice with 500 μl of nuclear lysis wash buffer [50 mM Tris (pH 7.0), 10 mM EDTA, 1% SDS, 1 mM DTT, 1 mM PMSF, protease inhibitors, and RNase inhibitor (0.1 U/μl)] and incubated with the chromatin at 37°C for 30 min. The cleared supernatant was separated magnetically and distributed into individual tubes for pull-down reactions, reserving 20 μl for RNA, DNA, and protein inputs. Biotinylated oligonucleotide probes (1 to 2 μl of 100 μM pool per reaction) specific to *LINC00662* and a *lacZ* control were added, followed by a 4-hour hybridization at 37°C.

In parallel, streptavidin beads (110 to 210 μl) were washed twice in nuclear lysis wash buffer, blocked with tRNA (500 ng/μl) and bovine serum albumin (1 mg/ml) for 1 hour at 37°C, and washed once again. Posthybridization, beads were added to the chromatin-probe mix and incubated for an additional hour at 37°C. The complexes were washed three times with wash buffer 1 [2× SSC, 0.5% SDS, 1 mM PMSF, 1 mM DTT, and RNase inhibitor (0.1 U/μl)] at 37°C, 500 rpm for 5 min each. After magnetic separation, residual buffer was removed by brief centrifugation.

For protein recovery, 100 μl of biotin elution buffer (7.5 mM Hepes, 12.5 mM biotin, 0.15% SDS, 0.075% sarkosyl, 0.02% sodium deoxycholate, 75 mM NaCl, and 1.5 mM EDTA) was added and incubated for 20 min at room temperature with agitation, followed by 10 min at 65°C. The eluate was collected magnetically, and the process was repeated once for a final volume of 200 μl, reserving 4 μl (2%) for RNA RT-PCR analysis.

For RNA isolation, both input and bead-bound fractions were treated with elution buffer and proteinase K, incubated at 50°C for 50 min to reverse cross-links, and purified using TRIzol followed by Zymo RNA Clean & Concentrator columns (Zymo Research, R1014). TURBO DNase digestion was performed to eliminate any residual genomic DNA, and enrichment of target transcripts was confirmed via RT-PCR.

#### Mass spectrometric analysis

Mass spectrometric analysis was carried out using an Orbitrap Fusion Tribrid instrument coupled to an Easy-nLC 1200 nano–liquid chromatography system (Thermo Fisher Scientific). Peptide samples were first loaded onto an Acclaim PepMap 100 C18 trap column (100 μm by 2 cm, 5-μm particle size; Thermo Fisher Scientific) and subsequently separated on an in-house packed analytical column (75 μm by 300 mm, 3 μm ReproSil-Pur C18, Dr. Maisch). A linear gradient from 5% to 28% acetonitrile (ACN) in 0.1% formic acid (FA) was applied over 75 min, followed by a rapid increase to 80% ACN in 0.1% FA for 5 min.

Full MS spectra were collected in the Orbitrap at a resolution of 120,000 within a mass/charge ratio (*m/z*) range of 375 to 1500. The most intense precursor ions (charge states 2 to 7) were selected for data-dependent fragmentation using higher-energy collisional dissociation (HCD) at a normalized collision energy of 30. The “top speed” duty cycle was maintained at 1 s, with an isolation window of 0.7 *m/z* and dynamic exclusion set to 10 parts per million (ppm) for 45 s. MS/MS scans were recorded at 30,000 resolution with a maximum injection time of 110 ms. Analytical blanks were run between samples to prevent carryover.

Raw data were processed using the Proteome Discoverer software (v2.4, Thermo Fisher Scientific). Peptide identification was performed with Mascot against the SwissProt human database, allowing one missed tryptic cleavage. Search parameters included precursor and fragment mass tolerances of 5 ppm and 30 mmu, respectively. Methionine oxidation and cysteine alkylation were set as variable modifications. False discovery rate (FDR) control was implemented using a decoy database search, applying a strict 1% threshold at the peptide-spectrum match (PSM) level. Identified proteins were grouped by shared peptide sequences to reduce redundancy and filtered at 5% FDR.

#### ChOP-MS analysis

All downstream analysis was done using R. Peptide counts in each replicate were subtracted by the corresponding values of *lacZ* control to remove background noise. Furthermore, the data were normalized by dividing the corrected counts by the total count in the respective replicate and scaled to 1000. On the basis of average of normalized values across the replicates, the top 45 proteins were analyzed using StringDb (v11.5; RRID:SCR_005223) (*97*). In addition, information on the subcellular location of the proteins were obtained from the Human Protein Atlas (RRID:SCR_006710; proteinatlas.org) (*98*–*100*).

### Generation of *LINC00662*-CRISPRi hNPCs, iPSCs, and unguided neural organoids

To silence the expression of *LINC00662* in hNPCs and iPSCs, we used the protocol described in Johansson *et al.* (*101*). Single guide sequences targeting near the TSS were designed according to the GPP Portal (Broad Institute). The guide sequences were inserted into a deadCas9-KRAB-T2A-GFP lentiviral backbone, pLV hU6-sgRNA hUbC-dCas9-KRAB-T2a-GFP, a gift from C. Gersbach (Addgene, plasmid #71237; RRID:Addgene_71237), using annealed oligos and the BsmBI cloning site. The lentivirus for each guide was produced as described above, and iPSCs were transduced with an MOI of 7.5 of *lacZ* or one of the *LINC00662*-targeting gRNAs (hNPCs were transduced with an MOI of 5).

*LINC00662* guide 1: CCCTGTGCGAGGTCTAACCC

*LINC00662* guide 2: TGCTCCATAGGAGGACCCAT

*lacZ*: TGCGAATACGCCCACGCGAT

#### GFP^+^ cell isolation of transduced iPSCs and hNPCs

Seven days after transduction, cells were detached with Accutase, resuspended in iPS/hNPC media containing Y27632 (10 μM) and Draq7 (1:1000), and strained with a 70-μm filter. Gating parameters were set by side and forward scatter to eliminate debris and aggregated cells. The GFP^+^ gates were set using untransduced iPSCs/hNPCs. The sorting gates were validated via reanalysis of sorted cells (>99% purity cutoff). A total of 300,000 GFP^+^/Draq7^−^ cells were collected per sample, spun down at 400*g* for 5 min, resuspended in iPS/hNPC media containing Y27632 (10 μM), and expanded as described above and frozen down for further use. Guide efficiency was validated using standard quantitative real-time RT-PCR.

#### LINC00662-CRISPRi unguided neural organoids

*LINC00662*-CRISPRi organoids were generated as described above for human iPSC lines (see “Unguided neural organoid culture”) with the exception that *LINC00662*-CRISPRi organoids were grown for 30 days. Two independent organoid batches were generated. Day 15 and day 30 organoids were collected for downstream analyses. Five day 15 and three day 30 organoids were collected and snap frozen for each snRNA-seq replicate (*n* = 2 per condition per batch). Five day 15 and three day 30 organoids were collected for immunostainings (see “Immunohistochemistry”) per condition per batch. Five organoids were collected for long-read DNA sequencing.

### Human tissue

Human fetal forebrain tissue was obtained from material available following elective termination of pregnancy at the University Hospital in Malmö, Sweden, in accordance with the national ethical permit (Dnr 2022-05051-01).
