# Supplementary material for: Retroviral insertions contributed to the divergence of human and chimpanzee brains
Source: Sci Adv. 2026 Oct 23;12(43):eaeg5791. doi: 10.1126/sciadv.aeg5791 (PMC13693685; doi:10.1126/sciadv.aeg5791)
Supplement: Supplementary file 1 [file sciadv.aeg5791_sm.pdf]

Science Advances
AAAS

Supplementary Materials for

Retroviral insertions contributed to the divergence of human and chimpanzee brains

Patricia Gerdes *et al.*

Corresponding author: Johan Jakobsson, johan.jakobsson@med.lu.se

Sci. Adv. **12**, eaeg5791 (2026)
DOI: 10.1126/sciadv.aeg5791

The PDF file includes:

Figs. S1 to S4
Tables S1 and S2
Legend for data S1

Other Supplementary Material for this manuscript includes the following:

Data S1

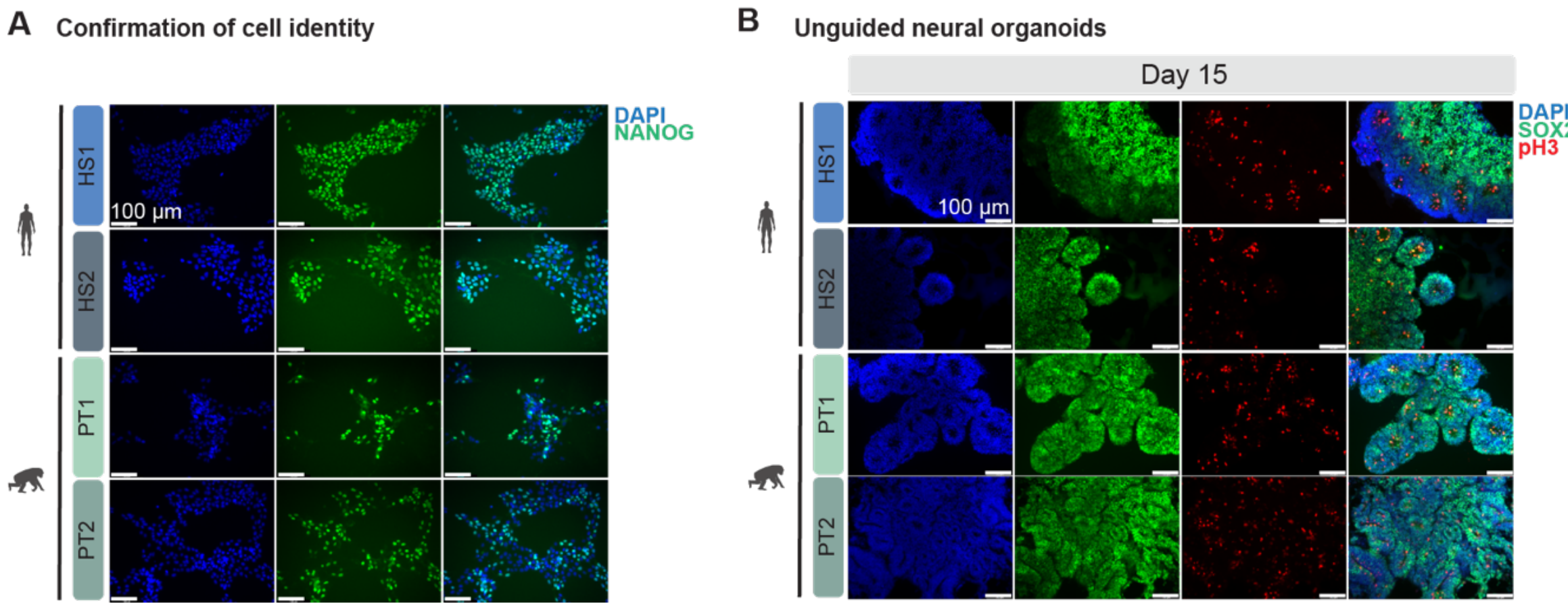

Fig. S1. Human and chimpanzee iPSCs and neural organoids show comparable pluripotency and early neural organization. A Immunocytochemistry of human and chimpanzee iPSCs. Pluripotent cells stained with NANOG (green), nuclei stained with DAPI (blue). Scale bars = 100 µm. **B** Representative immunofluorescence images of neural rosettes at day 15 of neural organoid differentiation, stained for neural progenitor cell marker SOX2 (green) and mitosis marker pH3 (red). DAPI is shown in blue. Scale bar, 100 µm.

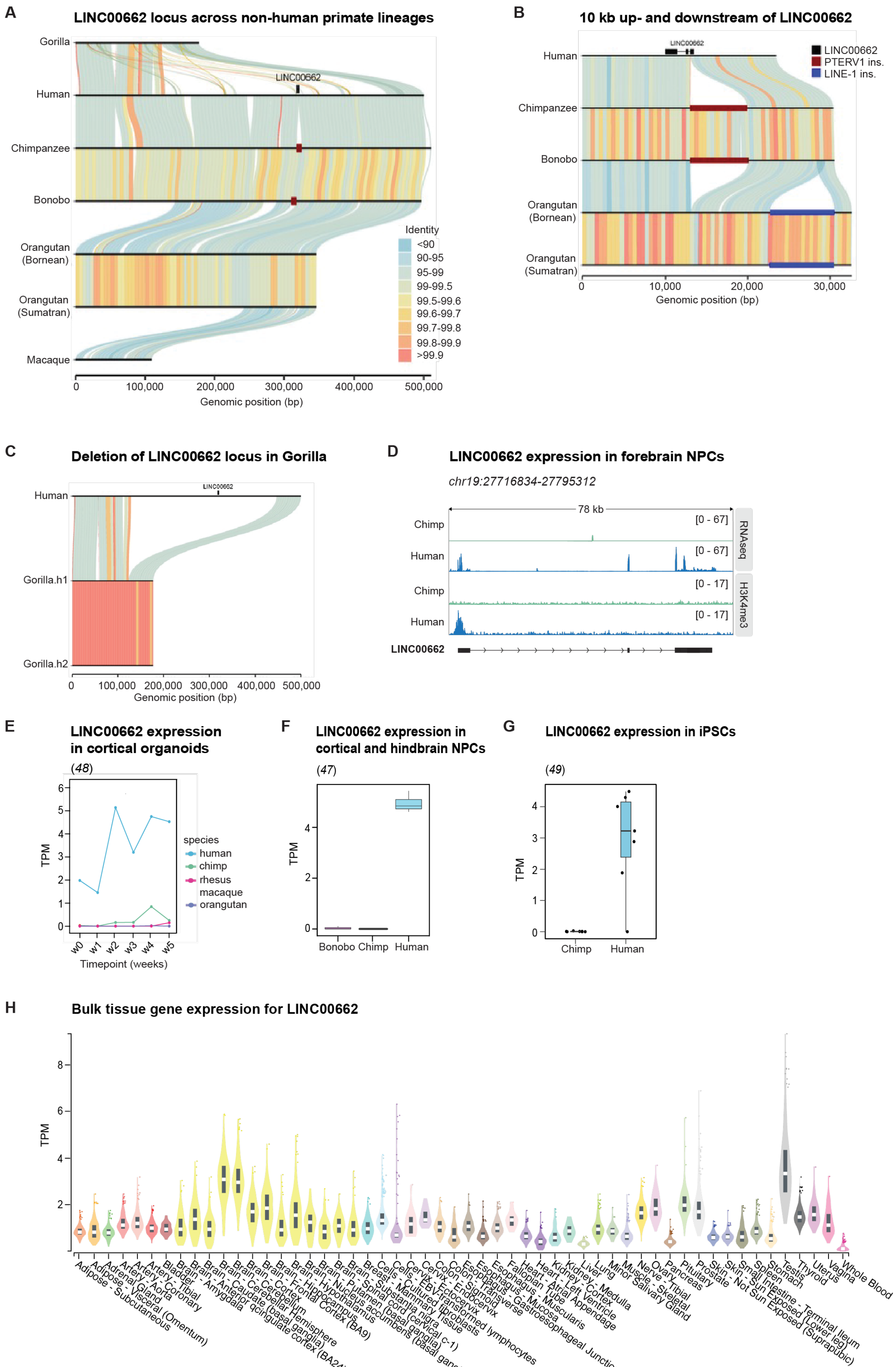
A
LINC00662 locus across non-human primate lineages
B
10 kb up- and downstream of LINC00662
C
Deletion of LINC00662 locus in Gorilla
D
LINC00662 expression in forebrain NPCs
chr19:27716834-27795312
E
LINC00662 expression in cortical organoids
(48)
F
LINC00662 expression in cortical and hindbrain NPCs
(47)
G
LINC00662 expression in iPSCs
(49)
H
Bulk tissue gene expression for LINC00662

Fig. S2. Comparative genomics and transcriptomic datasets support human-specific LINC00662 expression and chimpanzee-specific PTERV1 insertion. A Stacked alignment of genomic segments containing the *LINC00662* locus in human and NHP telomere-to-telomere (T2T) assemblies. Lines indicate homology between the human reference genome (T2T-CHM13) and gorilla (NCBI: PRJNA942267), chimpanzee (NCBI: PRJNA916736), bonobo (NCBI: PRJNA942951), Bornean orangutan (NCBI: PRJNA916742), Sumatran orangutan (NCBI: PRJNA916743), and rhesus macaque (T2T-MFA8v1.1). The PTERV insertion is present in chimpanzee and bonobo and is shown in dark red. **B** Zoomed-in view of 10 kb upstream and downstream of *LINC00662*. The *LINC00662* gene model is shown as a black track and is in the inverted orientation. The PTERV1 insertion in chimpanzee and bonobo is indicated in red, and an upstream LINE-1 insertion in orangutan is shown in blue. **C** Deletion of the segment containing *LINC00662* in two gorilla haplotypes. **D** Genome browser snapshot of *LINC00662* locus in hg38. Top two tracks show bulk RNA-seq data from chimpanzee and human fbNPCs respectively. Bottom two tracks showing H3K4me3 CUT&RUN data of chimpanzee and human fbNPCs respectively. **E** Transcripts per million (TPM) of LINC00662 in human (blue), chimpanzee (green), rhesus macaque (pink), and orangutan (purple) cortical organoids as reported by Field et al. (*48*). **F** TPM of LINC00662 in human (blue), chimp (green) and bonobo (purple) in cortical and hindbrain NPCs as reported by Marchetto et al. (*47*). **G** TPM of LINC00662 in human (blue) and chimp (green) in iPSCs as reported by Gallego et al. (*49*). **H** GTEx expression profile of LINC00662 across human tissues.

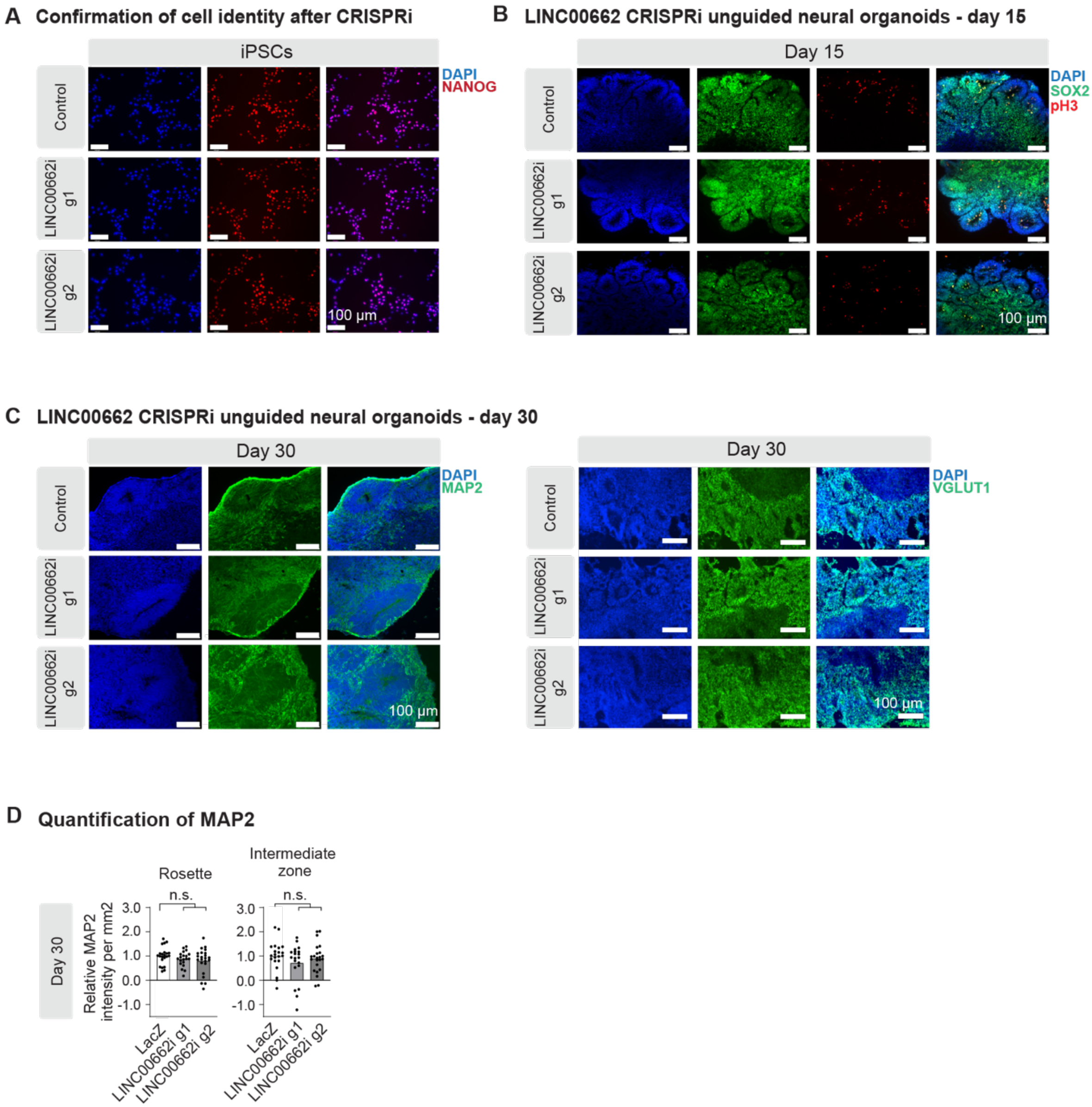

Fig. S3. LINC00662-CRISPRi organoids retain neural identity but show altered neuronal maturation markers. A Immunocytochemistry of *LINC00662*-CRISPRi iPSCs. Pluripotent cells stained with NANOG (red), nuclei stained with DAPI (blue). Scale bars = 100 µm. **B** Representative immunofluorescence images of neural rosettes in *LINC00662*-CRISPRi unguided neural organoids at day 15 of neural organoid differentiation, stained for neural progenitor cell marker SOX2 (green) and mitosis marker pH3 (red). DAPI is shown in blue. Scale bar, 100 µm. **C** Representative immunofluorescence images of *LINC00662*-CRISPRi unguided neural organoids at day 30 of neural organoid differentiation, stained for neuronal marker MAP2 (green, *left*) and glutamatergic neuronal marker VGLUT1 (green, *right*). DAPI is shown in blue. Scale bar, 100 µm. **D** Quantification of normalized MAP2 staining intensity in regions of interest, including 2–3 rosettes and intermediate zones per organoid from different planes, across six organoids per condition from two independent batches of day 30 LINC00662-CRISPRi organoids.

Two-tailed Student's t test. Each datapoint represents quantified region from an individual organoid slice.

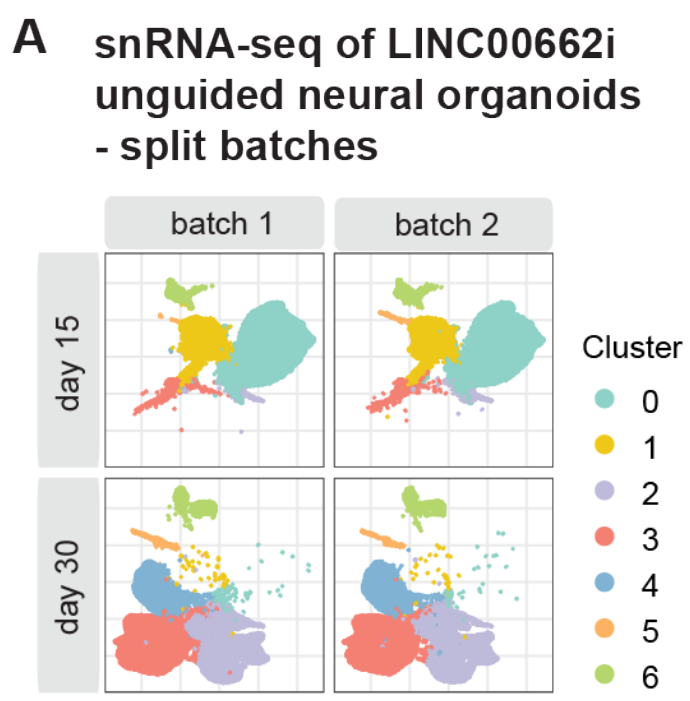

Fig. S4. snRNA-seq analysis confirms developmental-stage separation without major batch-driven clustering in LINC00662-CRISPRi organoids. A UMAP embeddings of nuclei from day 15 and day 30 *LINC00662*-CRISPRI unguided neural organoids, showing clustering of all samples and separation by differentiation time point (day 15 and day 30) and batch (batch 1 and batch 2).

Table S1. Differentially expressed genes overlapping PTERV1 insertions in chimpanzee (panTro6).

Gene name	Gene annotation (panTro6)	Human ortholog (hg38)	PTERV1 insertion (panTro6)	PTERV1 length (bp)	Overlap type
RP11-379B18.9	*LOC107970747*	*ENSG00000287617.1*	chr3:123907427-123915062	7635	intronic
TGFA	*LOC107973510*	*ENSG00000163235.16*	chr2A:70555032-70563195	8163	intronic*
AOAH	*AOAH*	*ENSG00000136250.14*	chr7:36726803-36735167	8364	intronic
GABRB1	*GABRB1*	*ENSG00000163288.14*	chr4:79972004-79980404	8400	intronic
AC004538.3	*LOC104007110*	*ENSG00000230333.6*	chr7:11263452-11271213	7761	intronic*
LINC00662	*LOC740918*	*ENSG00000261824.7*	chr19:25706377-25713292	6915	intronic
CTC-459F4.3	*LOC101927151*	*ENSG00000267575.7*	chr19:25706377-25713292	6915	UTR*
THSD7A	*LOC104007110*	*ENSG00000005108.16*	chr7:11263452-11271213	7761	intronic
LRP1B	*LRP1B*	*ENSG00000168702.18*	chr2B:27884750-27892948	8198	intronic
SNHG31	*LOC112208398*	*ENSG00000229267.3*	chr2B:101475274-101483109	7835	intronic

*Genomic context (e.g., intronic, UTR, downstream) is based on UCSC RefSeq annotations for panTro6 and may vary across annotation sets (e.g., Ensembl).

Table S2. Structural variants identified in LINC00662 locus (+/- 10 kb) across ape genomes.

Chromosome	Position	SV type	Size	Ref	Alt	PTR	PPA	PPY	PAB
chr19	30311503	DEL	56	CAAA...ACAG	C	1|1	1|1	0|0	0|0
chr19	30321123	INS	76	A	ATGA...GGCC	0|0	0|0	1|1	1|1
chr19	30321321	INS	7069	T	TTTA...TTCA	1|1	1|1	0|0	0|0
chr19	30321917	DEL	61	GTAC...AGGT	G	0|0	0|0	0|0	1|0
chr19	30321940	INS	80	T	TGGA...GTAC	0|0	0|1	0|0	0|0
chr19	30324693	INS	310	C	CAAG...AAAA	0|0	0|0	1|1	1|1
chr19	30329686	INS	8857	T	TGTA...GAGA	0|0	0|0	1|1	1|1

Chromosome, genomic position, variant type, size, reference allele, and alternative allele are shown for each structural variant (SV). Genotype calls are indicated for chimpanzee (PTR), bonobo (PPA), orangutan (PPY), and gorilla (PAB). Abbreviations: SV, structural variant; DEL, deletion; INS, insertion; Ref, reference allele; Alt, alternative allele; PTR, chimpanzee; PPA, bonobo; PPY, orangutan; PAB, gorilla.

Data S1. PTERV1 elements in panTro6 identified by RetroTector.
